# MicroRNAs and Stem-like Properties: The Complex Regulation Underlying Stemness Maintenance and Cancer Development

**DOI:** 10.3390/biom11081074

**Published:** 2021-07-21

**Authors:** Giuseppina Divisato, Silvia Piscitelli, Mariantonietta Elia, Emanuela Cascone, Silvia Parisi

**Affiliations:** Department of Molecular Medicine and Medical Biotechnology, University of Naples “Federico II”, via Pansini 5, 80131 Naples, Italy; giuseppina.divisato@unina.it (G.D.); silvia.piscitelli@unina.it (S.P.); maria.elia@studenti.unina.it (M.E.); ema.cascone@studenti.unina.it (E.C.)

**Keywords:** embryonic stem cells, cancer stem cells, microRNAs, noncanonical microRNAs, nuclear microRNAs, mirtrons, isomiRs, epithelial-to-mesenchymal transition, competitive endogenous microRNAs, circular RNAs

## Abstract

Embryonic stem cells (ESCs) have the extraordinary properties to indefinitely proliferate and self-renew in culture to produce different cell progeny through differentiation. This latter process recapitulates embryonic development and requires rounds of the epithelial–mesenchymal transition (EMT). EMT is characterized by the loss of the epithelial features and the acquisition of the typical phenotype of the mesenchymal cells. In pathological conditions, EMT can confer stemness or stem-like phenotypes, playing a role in the tumorigenic process. Cancer stem cells (CSCs) represent a subpopulation, found in the tumor tissues, with stem-like properties such as uncontrolled proliferation, self-renewal, and ability to differentiate into different cell types. ESCs and CSCs share numerous features (pluripotency, self-renewal, expression of stemness genes, and acquisition of epithelial–mesenchymal features), and most of them are under the control of microRNAs (miRNAs). These small molecules have relevant roles during both embryogenesis and cancer development. The aim of this review was to recapitulate molecular mechanisms shared by ESCs and CSCs, with a special focus on the recently identified classes of microRNAs (noncanonical miRNAs, mirtrons, isomiRs, and competitive endogenous miRNAs) and their complex functions during embryogenesis and cancer development.

## 1. Introduction

**Figure 1:**
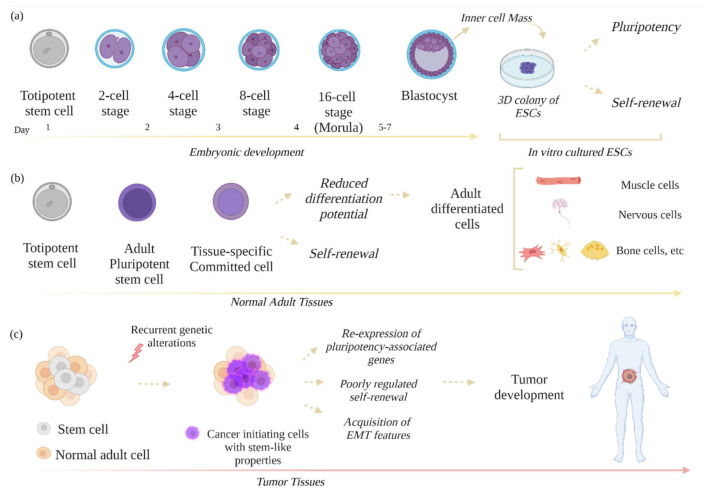
Schematic representation of different types of stem cells. (**a**) ESCs are derived from the inner cell mass of the blastocyst and can be cultured in vitro as colony-forming cells. (**b**) Adult pluripotent stem cells, derived from a totipotent cell, differentiate in tissue-specific committed cells, which contribute to tissue homeostasis and regeneration by forming adult terminally differentiated cells. (**c**) In pathological conditions, normal cells or stem cells can accumulate genetic mutations and can transform into cancer-initiating cells with stem-like features, which can take over the other cells, forming the tumor mass.

Embryonic stem cells (ESCs), adult stem cells, and cancer stem cells (CSCs) represent different typologies of stem cells that can be found in our body. ESCs are derived from the inner cell mass of the blastocyst and own the outstanding property of self-renewal, meaning that they can proliferate indefinitely, maintaining stem-cell characteristics [1]. ESCs are also classified as pluripotent cells since they can differentiate into all three germ layers of the embryo and their derivatives [2,3] (Figure 1a).

The balance between self-renewal and differentiation is governed by a multitude of transcription factors (TFs), signaling and epigenetic changes, and noncoding RNAs [4,5,6,7,8]. Adult stem cells, also known as tissue-specific stem cells, are undifferentiated cells located in different regions of our body. These cells have a limited self-renewal ability, with a reduced differentiation potential and a strong propensity to maintain and repair the tissue in which they reside [9] (Figure 1b). Therefore, they can be considered multipotent or unipotent stem cells. CSCs are a rare subpopulation of cells exhibiting stem-like features, and they are responsible for tumor initiation, development, and progression [10,11,12] (Figure 1c). The origin of CSCs is not still completely clear; they could be generated from tumor cells that acquire stem-cell properties or from normal stem cells that undergo a mutation during DNA replication [13,14]. CSCs can survive and take over the other cells inside the tumor mass, because they acquire exceptional properties that, in physiological conditions, are typical of ESCs [15]. These properties are shared by ESCs and CSCs, but not ESCs/non-CSCs, and they include pluripotency, self-renewal, expression of stemness genes, and acquisition of epithelial–mesenchymal features [16]. Specific biomarkers such as cell surface markers (CD133, CD15, CD44), intracellular molecules, and CSC transcription factors, detected by fluorescence-activated cell sorting (FACS) or other techniques, should be used to distinguish CSCs from other types of cells existing in the tumor mass [11,12]. Indeed, multiple levels of heterogenicity characterize the tumor tissue, which can contain cancer stem cells, cancer stem-like cells, and dedifferentiated cancer cells. In medulloblastoma and thyroid tumors, the cancer stem-like cell population represents a quiescent population inside the tumor, featuring an increased resistance to drug therapies and able to drive tumor heterogeneity, recurrence, and metastasis [17,18]. In some tumors, noncancer cells can even dedifferentiate into cells with a stem-cell phenotype, forming the dedifferentiated cancer cells [19,20]. Although the circumstances in which the dedifferentiation occurs are not completely clear, it has been reported that stress, wounding, or hypoxia can be responsible for this process [21]. Moreover, it has been hypothesized that the dedifferentiation of noncancer cells in cells with stem-cell phenotype could represent a further event for tumor initiation [21]. Stem-cell phenotypes in cancer can also be derived from paracrine effects from other cells; for example, endothelial cells can induce the CSC phenotype of human colorectal cancer cells by secreting factors promoting the CSC phenotype and Notch activation [22]. Similarly, the hypoxic microenvironment can promote the self-renewal ability of stem and non-stem cells, as well as stem-like phenotypes in non-stem populations, leading to glioma tumorigenesis [23]. CSCs can even differentiate in other cells, but following an abnormal differentiation process, as in teratocarcinoma, medulloblastoma, and leukemia cells [24,25]. As previously mentioned, CSCs acquire stem-like properties through the re-expression of genes typically expressed in ESCs such as *Nanog, Oct3/4*, and *Sox2*. In ESCs, the homeobox transcription factor (TF) *Nanog* maintains the pluripotency and establishes the proper ESC identity; the transcription factor *Oct3/4* is essential for pluripotency maintenance; the transcription factor *Sox2* is essential to stabilize ESCs in a pluripotent state acting synergistically with *Oct3/4* to regulate the expression of the pluripotent stem cell-specific genes [26,27,28,29]. These TFs act all together, generating a core pluripotency complex, crucial for ESC stemness. On the other hand, in CSCs, they drive cancer progression and are considered tumor biomarkers with prognostic value [30,31,32,33,34]. The molecular mechanisms commonly shared among ESCs and CSCs are numerous and often complicated. Recently, microRNAs (miRNAs) emerged as key regulators of stemness, pluripotency maintenance, self-renewal control, differentiation, and epithelial-to-mesenchymal transition in both ESCs and CSCs [4,35]. MiRNAs are small endogenous single-stranded noncoding RNA molecules able to modulate gene expression at the post-transcriptional level [36]. In their mature form, they bind target mRNAs by base pairing their seed sequence to a region located in the target 3′ untranslated region (3′-UTR). This binding leads to repression of gene expression by inhibiting mRNA translation and/or promoting its degradation. Some families and clusters of microRNAs are highly expressed in ESCs and regulate different functions such as cell-cycle progression, pluripotency, self-renewal, metabolism, and early differentiation [4,37]. ESC-specific cell-cycle-regulating miRNAs also regulate the mechanisms underlying cancer progression and resistance to the pharmacological treatment [35,38,39,40]. Likewise, the LIN28/*let-7* axis, which in ESCs is responsible for the selective block of miRNAs belonging to *let-7* family, is altered in CSCs [41,42,43]. In fact, in many cancers, high expression of the RNA-binding protein LIN28 is responsible for a global post-transcriptional downregulation of *let-7*, leading to an increase in different oncogenic targets (MYC, RAS, HMGA2, and others) and promoting tumorigenesis and cancer progression [43]. These and many other findings point out that the molecular mechanisms governing the stemness of CSCs are similar to those occurring in ESCs. The aim of this review was to explore the molecular mechanisms shared by ESCs and CSCs, with a special focus on the most recent and complex functions orchestrated by miRNAs in both these contexts.

## 2. Noncanonical microRNAs Orchestrate New and Complex Functions in ESCs and CSCs

**Figure 2:**
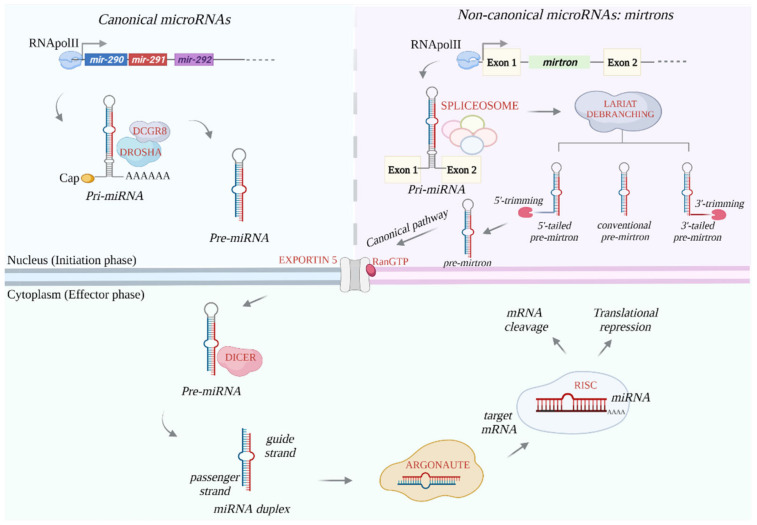
Biogenesis of canonical and noncanonical microRNAs. In the top left panel, the initiation phase of canonical miRNA biogenesis is summarized. The top right panel shows the initiation phase of noncanonical miRNAs (mirtrons), whose processing occurs in a Drosha-independent manner and requires the action of spliceosome and debranching enzymes in the nucleus. In the cytoplasm (bottom panel), both canonical and noncanonical miRNAs undergo the same effector phase.

The biogenesis of microRNAs is a mechanism that has been widely described for several decades. It is well accepted that miRNA biogenesis starts with transcription of a long primary transcript, called a pri-miRNA, that, in the canonical pathway, is processed in the nucleus by Drosha and DCGR8 enzymes (forming the microprocessor complex) and converted into a shorter transcript, called pre-miRNA, after a stem-loop cropping [44] (Figure 2).

Inside the cytoplasm, the pre-miRNA transcript is further processed by the endonuclease Dicer, which generates a small RNA duplex intermediate (about 22 nucleotides) [45]. The latter, together with the Argonaute (AGO) proteins, forms the RNA-induced silencing complex (RISC), which incorporates one strand of miRNA duplex as a template to complementarily bind a region in the 3′-UTR of the target mRNAs. The binding is mediated by a conserved heptametrical sequence, named seed sequence, typically spanning nucleotides 2–7 at the 5′-end of the microRNA sequence [46]. MicroRNAs work to finely regulate gene expression essentially in all cell processes. Many miRNAs are commonly expressed in ESCs and CSCs, but they fulfill context-specific functions (Table 1).

*MiR-200c* is highly enriched in human ESCs (H9 and HUES6 Wicells), where it regulates self-renewal and differentiation, while, in human pancreatic cancer stem cells, isolated from PANC-1 by FACS and expressing CD24, CD44, and ESA markers, it appears as an important EMT regulator [47,52]. *MiR-451* is upregulated in differentiating mESCs (E14Tg2a) and it is also involved in multiple cancer types (early-stage breast cancer, follicular thyroid tumor, lung adenocarcinoma, multiple myeloma, etc.), representing a biomarker for early cancer diagnosis and a therapeutic candidate for cancer treatment [57,59,60,61]. Although, for several years, the function of miRNAs has been linked to a simple repression of target mRNAs, some studies have indicated that they can orchestrate more complex mechanisms of gene expression regulation [113].

Recently, different noncanonical miRNAs were identified that fulfill roles in ESCs and CSCs. Table 2 summarizes some examples of noncanonical miRNAs acting in both contexts.

Noncanonical miRNAs resemble the structure and function of canonical miRNAs, but they undergo a different maturation pathway, for example in the initiation phase inside the nucleus [125,126] (Figure 2). Examples of noncanonical miRNAs are the alternative precursors named mirtrons, encoded by introns located in the coding regions of some genes, whose pre-miRNAs are generated by intron splicing machinery, bypassing Drosha processing [127] (Figure 2). There are three classes of splicing-derived miRNAs in mammals: conventional mirtrons, 5′-tailed mirtrons, and 3′-tailed mirtrons [128]. In conventional mirtrons, both ends of the pre-miRNA hairpin are defined by splicing mechanisms; they are exported in the cytoplasm and enter in the canonical pathway at level of Exportin 5 [128,129]. The 5′-end hairpins of 3′-tailed mirtrons feature the 5′-splite site, while the 3′-end features the branch point; in 5′-tailed mirtrons, the hairpin at the 3′-end of the intron is preceded by an unstructured region [130,131]. This indicates that tailed mirtrons have only one end of the pre-miRNA, which is formed by the splicing machinery. Of interest, Dicer recognizes and processes the 3′-end of 5′-extended pre-miRNAs, generating mature 3p and extended 5p miRNAs [132]. The analysis of deep sequencing data of small RNA sequences revealed that some miRNAs, annotated as canonical miRNAs in the available miRNAs databases, are processed through a noncanonical pathway [133]. Dicer processing has been reported as a mechanism almost essential for the biogenesis of both canonical and noncanonical miRNAs, while Drosha/DCGR8 cleavage has a more limited role. Indeed, while *Dicer* knockout caused the loss of canonical and noncanonical miRNAs, in *Dcgr8/Drosha* KO ESCs, the biogenesis of noncanonical miRNAs was preserved [134,135]. High-throughput sequencing data obtained from *Dgcr8* wild-type, *Dgcr8* KO, and *Dicer* KO cells revealed that mESCs express noncanonical miRNAs, as confirmed later by other studies [70,115,116].

Another class of noncanonical miRNAs is represented by small nucleolar RNA (snoRNA)-derived miRNAs. They are small noncoding RNAs, localized in the nucleolus, with a role in ribosomal RNA (rRNA) biogenesis and in the chemical modification of rRNA [136]. SnoRNA-derived miRNAs can regulate transcription or can bind the 3′-UTR of the target mRNAs, inhibiting their expression and functioning as miRNAs [137,138]. The analysis of short RNAs sequencing data also led to the identification of a new type of non-canonical miRNAs, represented by transfer RNA (tRNA)-derived miRNAs, important regulators of protein translation [139,140]. This new class of noncoding RNAs shares functional features with microRNAs; they undergo Dicer1 processing, form RISC complexes with Argonaute proteins and repress the expression of their target mRNAs [141]. Additional proteins able to generate tRNAs are RNase Z, which produces tRNAs from premature tRNA transcripts, and Angiogenin, which makes tRNAs under stress conditions. Thus, tRNA-processing enzymes, such as RNase Z, could generate functional miRNA-like species [125]. The 5′-tRNA-derived small RNAs can modulate the stem-cell state of mESCs [142]. Some 5′-tRNA-derived small RNAs, upregulated during mESC differentiation, interact with the RNA-binding protein IGF2BP1, leading to the repression of the pluripotency promoter factor *c-Myc*. tRNA-derived miRNAs have also been well characterized in the cancer context, where they share functional characteristics with microRNAs, repressing mRNA transcripts in a sequence-specific manner [141,143]. Aberrantly upregulated noncanonical miRNAs are responsible for the maintenance of malignant properties of CSCs that express low Drosha levels [119]. Altogether, this evidence revealed that noncanonical miRNAs orchestrate important functions in stem cells, and their deregulation is linked with the development of pathological states that often flow in cancer development.

## 3. Nuclear Functions of microRNAs

In the canonical pathway, the mature miRNAs promote translation inhibition of their target mRNAs through RISC in the cytoplasm [144,145]. However, different studies demonstrated that miRNAs can also act in the nucleus. The presence of microRNAs inside the nucleus is justified by the presence of some components of the miRNA pathway in this location. For example, the nuclear miRNA pathway contemplates the presence of Argonaute 2 and catalytically active Dicer in the nucleus. In this district, miRNAs are bound to the AGO2 protein, but the loading of duplex RNA is missing, and components of RISC are absent, indicating that the machinery is different between the nucleus and cytoplasm [146]. Different sizes between cytosolic and nuclear RISC were found; while a large complex has been detected in the cytoplasm (approximately 3 MDa), a smaller complex, only formed by AGO2 and a short RNA, has been identified in the nucleus (158 kDa) [147]. Therefore, it is hypothesized that the AGO2/miRNA complex could be formed outside the nucleus. Then, the formed minimal RISC complex could be imported in the nucleus [148]. The nuclear transport of microRNAs is facilitated by the presence of different nuclear localization signals (AGUGUU motif, consensus ASUS sequence, 5′–UUGCAUAGU–3′ and 5′–AGGUUGKSUG–3′ motifs) located in their sequence [149,150]. The nucleus–cytoplasm trafficking of mature microRNAs is also mediated by Exportin-1, which serves for the translocation of both mature miRNAs and Argonaute proteins in the nucleus [151,152]. Accumulating evidence has revealed that miRNAs can be shuttled from the cytoplasm to the nucleus, because they are involved in the regulation of biogenesis and function of different noncoding RNAs, included other miRNAs and long noncoding RNAs, as well as in the transcriptional activation or silencing of the target genes [150,153]. Data from cell fractionation and high-throughput sequencing even predicted the genomic DNA-binding sites for nuclear miRNAs, which could play a role in the regulation of transcription [154]. Lastly, microRNAs can enter the nucleus to be modified, interact with nuclear proteins, or participate in mechanisms responsible for chromatin remodeling [155]. For example, *miR-21*, a microRNA involved in regulation of pluripotency in ESCs (E14Tg2a.4 cell line, Bay Genomics), represents one of the first microRNAs detected in the nuclear and cytoplasmic extracts of HeLa cells [62,63]. This microRNA acts in the REST–*miR-21*–SOX2 axis in ESCs; *miR-21* directly targets *Sox2*, decreasing its expression and reducing mESC self-renewal. In the undifferentiated state of ESCs, *miR-21* is repressed by the transcriptional repressor REST, to avoid the loss of self-renewal. *Mir-21* is also highly expressed in cancer stem/progenitor cells (CSPCs), isolated from human ovarian teratocarcinoma PA1 cells by FACS, using an antibody directed against the stem-cell marker CD133 [65]. In CSPCs, *mir-21* could promote tumorigenesis using different mechanisms; it could regulate the self-renewal of progenitor cells, could produce growth factors, or could induce the dedifferentiation of non-progenitor cancer cells, all of which lead to an enrichment of the stem-cell population [64]. These effects were evaluated by functional assays such as sphere formation and experiments aimed at evaluating CD133 expression (cell sorting, qRT-PCR) [64,65]. *MiR-29b* is another example of a miRNA with nuclear localization; while *miR-29a* is mainly located in the cytoplasm, *miR-29b* showed a nuclear localization mediated by a hexanucleotide terminal motif (AGUGUU) in its 3′-UTR [156]. The *MiR-29* family contributes to early differentiation of ESCs (E14Tg2a cell line) by regulating the expression of TET1, the dioxygenase converting 5′-methylcytosine into 5′-hydroxymethylcytosine [66]. TET1 is highly expressed in undifferentiated mESCs; in this context, *mir-29* directly targets the *Tet1* transcript, causing the downregulation of the TET1 protein and promoting the upregulation of trophoblast lineage markers. In cancer, *miR-29* mainly functions as a tumor suppressor although some studies have described it as an oncogene [67]. In ovarian cancer, *miR-29* has been proposed as an important regulator of cancer metabolism. Indeed, *miR-29b* re-expression in ovarian cancer cells inhibits glycolysis and glucose metabolism, directly targeting *AKT2* and *AKT3* [67,68]. In melanoma cells (A375 and A375-STA1 wt cells), it mediates antiproliferative effects by downregulating CDK6, a regulator of G1/S phase [67,69]. Additional examples of microRNAs with nuclear localization are *miR-320*, *miR-30e*, and *miR-122* as well as many human miRNAs identified in the nucleus of neural stem cells [157,158,159,160]. *MiR-320* and *miR-702* represent two noncanonical miRNAs able to induce the proliferation of *Dcgr8*-deficient ESCs (germline-competent wild-type (W4), *Dgcr8*-deficient (*Dgcr8*^Δ/Δ^), and *Dicer*-deficient (Dicer^Δ/Δ^) cells). These miRNAs bind the 3′-UTR region of the cell-cycle inhibitors *p57* and *p21*, unlocking the cells from the G1 cell-cycle arrest [70]. In cancer cells, *miR-320* has a tumor-suppressor function; it is downregulated in different cancers such as breast cancer, glioma, gastric cancer, retinoblastoma, and human non-small-cell lung cancer, and it represents an important EMT inhibitor by reducing the levels of E-Cadherin and increasing those of N-Cadherin and Vimentin [31,72,75,76,77,78]. *MiR-30* is one of the four miRNAs able to repress the expression of LIN28 protein in both ESCs (mouse embryonic stem cell lines R1 and C57BL/6J-693, ATCC and The Jackson Laboratory) and LIN28-positive human breast cancer cell line T47D, directly binding its 3′-UTR. This mechanism, finely regulated in ESCs, could cause the reactivation of LIN28 in cancer [79]. *MiR-122* is a regulator of ESC differentiation; it acts in the *miR-122*/*FoxA1*/*HNF4a*-positive feedback loop, and its overexpression promotes the hepatic differentiation of mESCs (Cyagen company, Santa Clara, CA, USA) [80]. *Mir-122* is expressed at low levels in hepatocellular carcinoma, and its expression inversely correlates with that of the G9A histone methyltransferase, causing worst overall survival of patients [81].

## 4. Noncanonical Gene Targeting of microRNAs

The analysis of miRNA targetome provided substantial information regarding the miRNA–target interactome [161]. Different microRNAs can regulate gene expression using atypical targeting mechanisms. A recent study revealed that up to 60% of miRNA–target interactions in cancer cells occur via noncanonical seed pairing [162]. Noteworthy, miRNAs having a low GC content in their seed sequence use the noncanonical gene targeting as dominant mechanism for target recognition. However, noncanonical gene targeting does not significantly reduce target expression, as instead happens for the canonical pathway [163]. Noncanonical gene targeting of microRNAs can occur through “seed-like motifs”, which include seed sequences containing mismatches, deletions, or wobble pairings [161,164]. One example is represented by *miR-155.* The transcriptome-wide identification of *miR-155* targets revealed that approximately 40% of *miR-155*-dependent Argonaute binding does not require a perfect seed match [165]. *MiR-155* is expressed in ESCs (mouse ESCs CGR8 cell line, ECACC) and is gradually downregulated during ESC cardiac differentiation [82]. It also represents a well-known oncogenic miRNA, overexpressed in different cancer cells with stem-like properties, such as breast cancer [83]. Soft agar colony formation assay and tumor xenografts revealed that *miR-155* inhibition reduced the proliferation of the invasive cell line MDA-MB-231, while its overexpression coincided with the acquisition of stem-like properties, as confirmed by sphere-forming experiments [83]. Another example of noncanonical targeting is represented by the microRNAs *miR-134*, *miR-296*, and *miR-470*. These miRNAs target the coding region of ESC pluripotency genes *Nanog*, *Oct4*, and *Sox2* in mESCs (E14Tg2a, ATCC) instead of/further than their 3′-UTR [166].

IsomiRs, distinct isoforms generated by miRNA precursor arms, are also considered part of noncanonical gene targeting mediated by miRNAs. IsomiRs can be generated by alternative Drosha and/or Dicer processing of pri/pre-miRNA molecules or by post-transcriptional modifications induced by the nucleotidyltransferase [167,168]. An example of how isomiRs can promote noncanonical gene targeting is provided by *miR-124*. The processing of *pri-miR-124* generates two 5′ isomiRs, derived from seed sequences shifted by a single nucleotide, which act by regulating different transcripts, contained in overlapping targetomes [169]. Next-generation sequencing data obtained from hESCs (CyT49 (ViaCyte), H1, and H9 cell lines) led to the identification of some miRNAs, such as *miR-302*, whose stem loop generates different highly expressed isomiRs, with important roles in hESC self-renewal [170]. For example, in hESCs, an miRNA mimetic for *miR-302a-5p* caused a reduced expression of OTX2, while isomiR *302a-5p(+3)* decreased the expression of tuberous sclerosis protein 1 [170]. Dominant isomiRs are also expressed during different stages of hESC differentiation. In differentiating hESCs (H9, HSF1) and induced pluripotent stem cell lines (hIPS2, all from UCLA Stem Cell Core), editing sites in 24 different miRNAs and major-to-minor arm-switching events in 14 pre-miRNAs have been identified [171]. IsomiRs also play important roles in cancer. They have been used to distinguish different breast cancer subtypes and, thus, act as biomarkers [172]. IsomiR expression changes in gastric tumor tissues; for instance, the processing of the same pre-miRNA can generate differentially expressed 5p and 3p arm miRNAs: one specific for the normal tissue, the other one specific for the tumor tissue [173]. Collectively, these data demonstrate that the functions mediated by miRNAs, which are already very complex, present an additional level of complexity due to the possibility to act through noncanonical gene targeting.

## 5. The Stemness Properties of ESCs and CSCs Are Regulated by the Same miRNA Circuits

CSCs, having expression signatures that are specific to ESCs, have been identified in many human tumors (human epithelial, breast, and lung cancers) and mouse cancer models [174]. In ESCs (mouse J1 ES cell line), the specific gene expression signature is orchestrated by different modules such as the core module, which includes the core pluripotency factors, the PRC module, represented by polycomb complex factors, and the MYC-module, including MYC-related factors. ESCs and cancer cells share MYC-module activity, raising the hypothesis that cancer cells can reactivate the ESC gene signature [175]. Similarly, the self-renewal ability of leukemia stem cells is sustained by a transcriptional subprogram more like that of ESCs than adult stem cells [176]. In general, cancer cells (mouse lung squamous carcinoma cells, SqCLCs, and KLN205) and mESCs (Celprogen) are very similar to each other, but highly different from normal cells in terms of spectral variations in protein, lipid, carbohydrate, and nucleic acid components [177]. Histologically poorly differentiated tumors also share features with ESCs; in fact, they overexpress the ESC-specific genes *NANOG*, *OCT4*, *SOX2,* and *c-MYC* and repress the *Polycomb*-regulated genes [178]. Invasive breast cancers are also able to secrete embryonic morphogens such as NODAL, an embryonic molecule that guides the transition from naïve to formative pluripotency [179,180]. Recent studies demonstrated that microRNAs could regulate similar circuits in ESCs and CSCs. For example, *miR-302* is an ESC-specific miRNA that orchestrates the cell cycle, acting in the G1/S cell phase of *Dgcr8* knockout (*Dgcr8*^Δ/Δ^) ESCs [84]. *MiR-302* regulates human iPSCs tumorigenicity, suppressing the expression of Cyclin E-CDK2 and Cyclin D-CDK4/6, promoting the expression of the senescence-associated tumor-suppressor genes, and reducing G1–S cell-cycle transition [89]. In this section, we provide an overview of recently described microRNAs able to regulate common functions in ESCs and CSCs, with the purpose of highlighting the molecular mechanisms linking these two faces of stemness.

### 5.1. Pluripotency-Regulating miRNAs in ESCs and CSCs: The Role of LIN28/let-7 Axis

Different clusters and families of microRNAs regulate the stemness and pluripotency of ESCs. Two major clusters, represented by *mir-290-295* and *mir-302-367*, are highly expressed in ESCs (J1 ESCs and induced pluripotent stem cell lines), where they regulate cell-cycle progression and are responsible for the induction of stemness properties [4]. These clusters are downstream targets of the pluripotency transcription factors [85]. OCT4 and SOX2 bind a conserved region in *mir-302* promoter, such that its expression follows *Oct4* expression during embryogenesis. A similar mechanism occurs in P19 mouse embryonic carcinoma cells, where OCT4 is required for the expression and transcriptional activation of *mir-302*. Indeed, OCT4 binds the putative promoter of *mir-302*, activating the transcription of the primary *mir-302* [87]. Mechanistically, in human pluripotent stem cells (hESC lines H9 from Wicell and chHES-22), *miR-302* acts as a pluripotency inducer; this miRNA binds the 3′-UTR of target mRNAs involved in differentiation processes, such as *AKT1*, and suppresses their expression, thus maintaining high levels of OCT4 and hampering teratoma formation [88]. However, in human glioma-initiating cells (primary cell lines TG1, TG6, and GB1, isolated from human glioblastoma), *miR-302* has an opposite effect, because it promotes the exit from the stem-cell-like state. Indeed, its endogenous expression is inversely correlated with NANOG and OCT4, and its ectopic expression in glioma stem cells is sufficient to repress OCT4 and NANOG, as well as tumor aggressiveness [39]. In detail, the suppression of the stem-cell-like signature induced by the *miR-302-367* cluster in glioma-initiating cells is mediated by the drastic downregulation of the pathway mediated by the chemokine receptor CXCR4 and its ligand SDF1, because *miR-302a* binds to the *CXCR4* 3′-UTR. This pathway, when suppressed, causes the disruption of the network SHH–GLI–NANOG responsible for the acquisition of the stem-cell-like signature, inhibiting the tumorigenic properties of the glioma-initiating cells [39]. The *MiR-302-367* cluster also causes a pronounced downregulation of transcripts normally involved in the regulation of cell-cycle progression, such as *E2F1*, *cyclin A*, and *cyclin D*, revealing that its expression had a negative impact on cell infiltration and self-renewal. These observations indicate that, in the cancer context, *miR-302* can play a dual role, because, as discussed in the previous section, it can also promote the expression of the senescence-associated tumor-suppressor genes [89].

**Figure 3:**
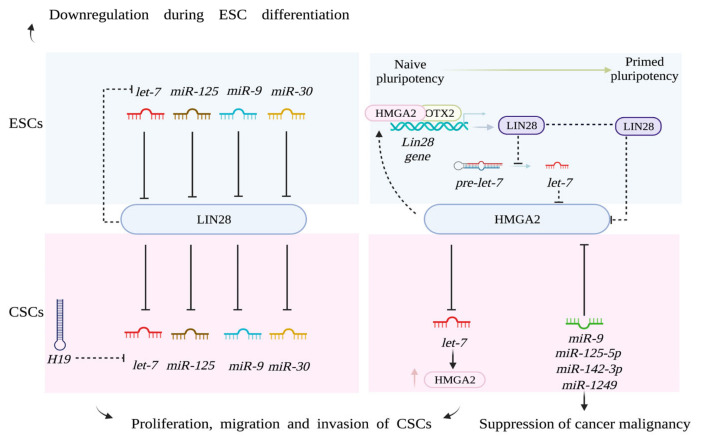
MicroRNAs regulating the onco-embryonic proteins LIN28 and HMGA2 in ESCs and CSCs. In the left panel (upper section), microRNAs repressing *Lin28* during embryogenesis are reported. In CSCs, this regulation is completely reverted, because the oncogenic protein LIN28 represses the expression of the same miRNAs (lower section of the left panel) causing tumorigenesis. In the right panel (upper section), the mechanism responsible for the transient induction of LIN28 upon ESC exit from the naïve ground state of pluripotency is described. This event is promoted by HMGA2-dependent engagement of OTX2 to *Lin28* enhancers. In undifferentiated ESCs, LIN28 represses *let-7* biogenesis, while, during the differentiation, it controls the levels of *Hmga2*, binding a conserved region in its 3′-UTR (let-7-independent mechanism). In the lower section of the right panel, the miRNA/HMGA2 axes acting in CSCs are described; the inhibition of *let-7* miRNAs by HMGA2 causes proliferation, migration, and invasion of CSCs, while the repression of *Hmga2* expression by specific microRNAs suppresses cancer malignancy.

Pluripotency in ESCs and CSCs is regulated by the overlapping functions of miRNAs and RNA-binding proteins. An example is provided by the RNA-binding protein LIN28. In physiological conditions, LIN28 expression is highly restricted to ESCs (E14Tg2a cell line, Bay Genomics), where it is transiently induced after the exit of ESCs from the naïve ground state and downregulated in differentiated cells [42,181]. LIN28, together with OCT4, SOX2, and NANOG, can also reprogram somatic cells into induced pluripotent stem cells, functioning as important regulator of pluripotency [182,183]. In pathological conditions, it acts as potent oncogene, causing malignant transformation and tumor progression [184,185]. Indeed, LIN28 has a widely recognized role in cancer, where it promotes proliferation, migration, and invasion of human bladder cancer cell lines (5637, SW780, T24, and J82), using the same mechanisms described in mESCs [186]. Indeed, it can act as pluripotency inducer by regulating the biogenesis of microRNAs belonging to *let-7* family (Figure 3).

LIN28 inhibits the maturation of *let-7* miRNAs to maintain the cells in the undifferentiated state; therefore, its inhibition could represent a possible mechanism to block tumor progression [91,185]. As an RNA-binding protein, LIN28 recognizes and remodels stable planar structures of four guanines, known as G-quartet structures, which are found in microRNAs and its target mRNAs [187]. During embryogenesis, LIN28 levels are regulated by microRNAs such as *let-7*, *miR-125*, *miR-9* and *miR-30*, whose expression inversely correlates with LIN28 expression [79] (Figure 3). This negative correlation could explain the oncogenic function of LIN28; indeed, human breast cancer cell lines, expressing high levels of LIN28, are characterized by the downregulation of these miRNAs [79]. A recent study revealed that the treatment with a LIN28 inhibitor (small compound C1632) can increase the levels of *let-7* and suppress the expression of the immune checkpoint protein PD-1/PD-L1, reactivating the antitumor activity in MCF-7, U2OS, and HeLa cancer cells [188]. Moreover, *let-7* miRNAs can act as tumor suppressors, since, in non-small-cell lung cancer A549 cells harboring mutant KRAS, their substitution reduced both the stem-cell population and the resistance to chemotherapy, causing cytotoxicity and inducing apoptosis and reduced invasiveness of the tumor cells [92,93]. In some types of cancers (i.e., non-small-cell lung cancer), the reduced expression of *let-7*, caused by irradiation, is responsible for the increased expression of LIN28, which further decreases *let-7* levels, thereby promoting resistance to irradiation [189]. Indeed, in cancer cells, the downregulation of microRNAs belonging to the *let-7* family is a mechanism frequently adopted to promote tumor progression. For example, the long noncoding RNA *H19*, normally expressed during ESC differentiation, competitively sponges *let-7* microRNAs in breast cancer cells (MDA-MB-231 and SK-BR-3 cells), causing an increase in LIN28 expression. The latter further blocks *let-7* biogenesis and, in turn, derepresses *H19* expression, forming a double-negative feedback loop that promotes breast cancer stem-cell maintenance [94]. In multiple myeloma, the LIN28B/*let-7* axis modulates the expression of MYC, which in turn is a *let-7* target, suggesting a novel mechanism for therapeutic targeting of the tumor [95]. However, in rare cases, microRNAs belonging to the *let-7* family can also act as oncogenes, increasing cancer migration, invasion, and progression [92]. For example, an anchorage-independent assay revealed that *let-7a3* overexpression in lung cancer cells caused an increase in aggressiveness [96]. Similarly, *let-7f-5p* and *let-7e-5p* were highly expressed in oral cavity squamous cell carcinomas, with *let-7f-5p* upregulated in nonaggressive tumors and *let-7e-5p* in aggressive ones [97]. Some tumors are also characterized by LIN28 loss. For example, in glioblastoma stem cells LIN28 is undetectable, while *let-7* miRNAs and their targets are expressed. In this context, another RNA-binding protein, named insulin-like growth factor 2 mRNA-binding protein 2 (IMP2), binds to *let-7* miRNAs, preventing the repression of *let-7* targets [190].

### 5.2. Pluripotency-Regulating miRNAs in ESCs and CSCs Targeting the Architectural Protein HMGA2

In neuroendocrine pancreatic cancer cells, LIN28 can induce the stem-like genes, suppressing *let-7* miRNAs and derepressing HMGA2 [191]. HMGA2 is an architectural protein, expressed early during embryogenesis and in ESCs (E14Tg2a cell line, Bay Genomics), whose suppression hampers the exit of ESCs from the pluripotent ground state [6]. Interestingly in mESCs (E14Tg2a cell line Bay Genomics), Hmga2 is regulated by *let-7*-indipendent mechanisms. Indeed, Lin28a binds highly conserved elements located in Hmga2 mRNA to properly control Hmga2 accumulation during differentiation [42]. HMGA2 expression is undetectable in adult tissues, and it is significantly overexpressed in different cancers as hepatocellular carcinomas, esophageal squamous cell carcinoma, tongue squamous cell carcinoma, and thyroid carcinoma [192,193,194,195,196]. It represents another ESC-specific factor that can induce tumorigenesis using multiple mechanisms. For example, HMGA2 induces the proliferation of cancer cells (ovarian cancer, leukemia, breast cancer, and colorectal cancer) promoting cell-cycle entry and inhibiting apoptosis, but it can also exhibit effects on pathways involved in DNA repair and epithelial-to-mesenchymal transition [197,198,199,200,201]. These mechanisms are often mediated by different microRNAs. *HMGA2* 3′-UTR is directly targeted by *miR-142-3p*, inducing a decrease in HMGA2 protein and suppressing breast cancer malignancy [202]. *MiR-9* is another type of microRNA that mediates antitumor activities on hepatocellular carcinoma progression directly targeting *HMGA2* [203]. Similarly, *miR-1249* suppresses the growth, metastasis, and angiogenesis of colorectal cancer cells (HCT116, HT29, SW480, SW620, HCT8, and DLD-1) by targeting *VEGFA* and *HMGA2* [204]. M*iR-125b-5p* also inhibited cell proliferation, migration, and invasion of esophageal squamous cell carcinoma partially by downregulating *HMGA2* [205]. *HMGA2* 3′-UTR also contains repressive regulatory binding sites for *let-7* miRNAs, which are responsible for *HMGA2* mRNA decapping and degradation, allowing the correct tissue-type differentiation of the normal mesenchymal tissues [206]. The TRIM71 protein, which regulates early development and differentiation, can act as a tumor suppressor by post-transcriptionally repressing LIN28B and modulating the *let-7*/*Hmga2* axis [207]. The reduction in *let-7* miRNAs represents one of the main mechanisms responsible for HMGA2 overexpression in atypical teratoid/rhabdoid tumors; therefore, the reconstitution of *let-7* miRNA levels or *HMGA2* knockdown may represent good therapeutic strategies for cancer treatment [208]. In different mesenchymal tumors, chromosomal rearrangements and breakpoints can generate truncated *HMGA2* mRNA transcripts, which are devoid of the 3′-UTR regions, thus altering miRNA-mediated regulation of HMGA2 expression [206,209,210].

## 6. EMT-Regulating miRNAs in ESCs and CSCs

Epithelial-to-mesenchymal transition is a well-characterized process that can occur in physiological and pathological conditions such as embryonic development, tissue repair, wound healing, and cancer [50]. Typically, in EMT, the epithelial cells lose their typical traits and acquire mesenchymal features [211]. Epithelial cells are highly plastic cells, which, during embryogenesis and in certain physiological conditions, need to transiently repress their epithelial features (loss of epithelial junctions, repression of epithelial genes) and acquire mesenchymal properties (expression of mesenchymal genes, elongation of cell shape, migratory and invasive phenotype) [212]. This mechanism favors cell migration to a different location. During embryonic development, EMT allows the correct differentiation of cells and the remodeling of tissues. In the cancer context, this allows tumor cells to dissociate from the primary tumor mass and disseminate to distant organs. In the new location, the cells reactivate the epithelial features, causing metastasis [213]. EMT is a process initiated by paracrine signals produced by stromal cells, among which the most famous are the transforming growth factor (TGF)-β, Wnt, Notch, and Sonic Hedgehog [214,215]. All EMT factors are controlled at the transcriptional and translational level by transcription factors and microRNAs [216]. The EMT gene signatures and the relative interactomes (miRNAs, transcription factors, and proteins) are contained in a database, named EMTome, which can be used as a portal for research studies [213]. Many microRNAs regulate the expression of the EMT-transcription factors, such as *SNAIL1/SNAIL2*, *bHLH* (*E47*, *E2-2*, and *TWIST1/TWIST2*), and *ZEB1/ZEB2*, which mainly function as *E-Cadherin* repressors [217]. In hESCs (H9 and H1 cell lines, WiCell Institute), the zinc-finger E-box-binding homeobox (*Zeb*) transcription factor is targeted by *miR-200* family members, which are highly expressed in ESCs but downregulated in a Wnt-dependent manner during EMT [48,49]. During hESC differentiation (MC50 from Dr. Robert Schreiber and modified A2lox cells), the *miR-200* family downregulates the expression of the transcription factor *ZEB1* and its target *E-Cadherin*, to define the proper cell fate [49,50]. In ESCs, *miR-200* also acts against the transcription factor *Snail1* to regulate EMT [49]. Altogether, these mechanisms indicate that *miR-200* members have an inhibitory role in EMT, limiting it spatially and temporally. In the cancer context, members of the *miR-200* family are often repressed, such that EMT could occur without spatial and temporal limits. In fact, in colon cancer cells (Caco-2, LS174T, LoVo, HT-29, HCT116, SW480, and SW620) and human colorectal cancer tissues, *NANOG* directly represses the transcription of *mir-200b/c* genes, modulating EMT to mesenchymal–epithelial transition plasticity [53]. Similarly, *ZEB1* can promote tumor cell dissemination and metastasis, repressing the expression of the *miR-200* members [54]. Accordingly, a recent study revealed that *miR-200* removal in an insulinoma mouse model, as well as the depletion of *miR-200* sites in endogenous *Zeb1*, caused beta-cell dedifferentiation, EMT initiation, and tumor invasion [55]. *MiR-200* can also be sponged by lncRNAs, such as *lncATB*, which induces EMT, restoring TWIST1 expression and causing poor prognosis in breast cancer [56]. In breast tumors, *miR-200* family can exhibit a dual role; miRNAs belonging to this family have been found upregulated in breast tumors, while they are downregulated in more aggressive breast cancer molecular subtypes, revealing that the levels of *miR-200* members are correlated with the nature of the tumors [218]. Another important cluster of microRNAs regulating EMT during embryonic development and cancer initiation is represented by a microRNA cluster on chromosome 19 (*C19MC*). This is the largest human microRNA cluster, which contains 46 miRNAs [219]. Bioinformatics analysis showed that some of the *C19MC* miRNAs share the “AAGUGC” seed sequence with members of the *miR-302-372* family and that their putative targets could be involved in reprogramming and apoptosis induction [220]. *C19MC* is expressed in placenta, ESCs, and cancer. The transcriptional activation of the entire *C19MC* cistron in human villous trophoblasts resulted in the suppression of EMT-related genes and in the induction of the OCT4 and FGF4 expression [98]. This large cluster of microRNAs is a transcriptional hallmark of different types of cancers (type A and AB thymomas, hepatocellular carcinoma, undifferentiated embryonal sarcoma of the liver, embryonal tumor with multilayered rosettes, etc.) and undergoes recurrent chromosomal breakpoints [99,100,101,102,103]. For example, rearrangements of the chromosomal band 19q13.4 are a typical cytogenetic feature of the thyroid adenomas, and the miRNA gene cluster *C19MC* is near the breakpoint region [221]. *MiR-429* is another EMT suppressor that acts during embryo implantation, by targeting a member of cadherin family, named *Pcdh8* [104]. *MiR-429* acts as a tumor suppressor in colorectal cancer, thanks to its direct binding to *HMGB3*, a strong oncogene overexpressed in cancer tissues [105]. As previously mentioned, high-mobility group proteins also play an important role in EMT. HMGA2 promotes EMT by activating specific signaling pathways, as MAPK/ERK, TGFβ/SMAD, PI3K/AKT/mTOR, NF-κB, and STAT3 [201]. The TGF-β molecule can induce EMT by promoting the expression of the embryonic protein HMGA2 that, together with SMADs, regulates different EMT transcription factors [222]. During EMT, HMGA2 promotes the binding of the *de novo* DNA methyltransferase 3A (DNMT3A) to the *Cdh1* promoter, inducing the hypermethylation and silencing of the tumor-suppressor E-Cadherin (CDH1); this causes tumor cell invasion [222]. In endometrial cancer, the overexpressed lncRNA *miR-210-HG* sponges *miR-337-3p/137*, increasing HMGA2 expression and modulating the malignancy of the tumor via TGF-β/Wnt pathway [223]. Collectively, these data indicate that EMT can be considered a further mechanism commonly shared by ESCs and CSCs. Interestingly, the same classes of microRNAs and proteins, which are normally expressed during embryonic EMT, can be reactivated in pathological conditions, contributing to cancer development and progression.

## 7. Regulation of miRNAs through Competitive Endogenous RNAs in ESCs and CSCs

The functions mediated by microRNAs can be regulated by a further class of RNAs, which emerged in recent years, called competitive endogenous RNAs (ceRNAs). These RNA molecules can influence the functions mediated by microRNAs, competing with them inside the cells and providing an additional mechanism of post-transcriptional regulation [224]. The ceRNA–miRNA network can act in both physiological (cell differentiation, regeneration mechanisms, and neural and muscle developmental processes) and pathological conditions (cardiovascular and neurodegenerative diseases, and cancer) [224,225]. The regulatory network mediated by ceRNAs is quite complex because they cross-regulate each other by sponging shared miRNAs [226]. In ESCs, lncRNAs function as ceRNAs to regulate the expression of mRNAs by competitively binding miRNAs. For example, the lncRNA *LINC-ROR* forms a feedback-loop with transcription factors and microRNAs in self-renewing hESCs (H1 and X-01 cell lines, Zhejiang University) to regulate ESC maintenance and differentiation [227]. *LINC-ROR* shares miRNA response elements with the core transcription factors *NANOG*, *OCT4*, and *SOX2* and it prevents the binding of these transcription factors to miRNAs (*miR-145*), inhibiting the suppression of their expression. Similarly, *lnc-NAP*, a lncRNA activated by the pluripotency factors NANOG, OCT4, and SOX2, could inhibit the effects mediated by *miR-139-5p*, impairing its binding to *Nanog* 3′-UTR and causing *Nanog* de-repression in mESCs (C57BL/6J ESCs and B6D2F1 iPSCs) and embryos [228]. Aberrations in the ceRNA network can lead to pathological conditions and cancer development [229]. *LINC-ROR,* acting as a ceRNA in ESCs, is involved in the occurrence and development of different human tumors (breast cancer, colorectal cancer, pancreatic cancer, hepatocellular carcinoma), and it represents a potential biomarker with clinical significance that can be used as therapeutic target [230]. In colon cancer stem cells, as in ESCs, *LINC-ROR* acts as a ceRNA to prevent *miR-145*-mediated suppression of *NANOG*, *OCT4*, and *SOX2* TFs to regulate cell proliferation and chemosensitivity [231]. *LINC-ROR* sponge activity against *miR-145* also leads to the derepression of *ZEB2*, inducing EMT in hepatocellular carcinoma and promoting metastasis [232]. In human gastric cancer cells (AGS and MGC-803 cell lines), *LINC-ROR* sponges *miR-212-3p*, promoting the proliferation, migration, and invasion of CSCs, as demonstrated by the CCK-8 assay, transwell assays, and a xenograft mouse model [233]. *LINC-ROR* can also function as a ceRNA for some members of the *let-7* family. MTT, wound healing, and matrigel invasion assays, as well as sphere-formation experiments, revealed that the sponge activity of *LINC-ROR* against *let-7* miRNAs contributed to the stem-cell properties of pancreatic cancer cells [234]. In retinoblastoma tissues, *LINC-ROR* is activated by H3K27 acetylation and sponges *miR-32-5p*, modulating EMT and regulating Notch signaling [235]. LncRNA *XIST*, expressed in differentiating ESCs (F1 2-1 ESC line), promotes metastasis and EMT in colorectal cancer, sponging *miR-125b-2-3p* [236,237]. Likewise, many lncRNAs involved in colorectal cancer act as ceRNAs and regulate the expression of the EMT transcription factors such as *ZEB1*, 2*/E-Cadherin*, and *Wnt/β-Catenin* signaling [238]. CeRNAs have been described as critical components of the TGFβ-induced EMT pathway, and they represent potential targets to disrupt EMT during tumorigenesis [226].

As previously mentioned for *LINC-ROR*, competitive endogenous RNAs also play important roles in CSCs. For example, the *actin filament-associated protein 1 antisense RNA 1* (*AFAP1-AS1*), functioning as an endogenous RNA, competitively binds to *miR-384*, regulating the expression of the Activin Receptor A type I (ACVR1) and inhibiting the stemness of pancreatic cancer cells and tumorigenicity in nude mice [239]. Similarly, the transcription factor *E2F6* can also function as a ceRNA, inhibiting the effects mediated by the tumor suppressor *miR-193a* and promoting the stemness of ovarian cancer cells (HeyC2) through the upregulation of the ovarian cancer stemness marker c-KIT [240]. In thyroid cancer cells (TPC-1 and K-1, ATCC), the long noncoding RNA *H19*, acting as a ceRNA, inhibits the effects mediated by *miRNA-3126-5*p, increasing the expression of the *estrogen receptor β* and inducing cancer stem-like properties [241]. Similarly, *H19* is responsible for glycolysis and maintenance of breast cancer stem cells thanks to its ability to bind to *let-7* miRNA, releasing *hypoxia-inducible factor 1α* and increasing the expression of pyruvate dehydrogenase kinase 1, protein highly expressed in breast cancer stem cells [242].

Circular RNAs (circRNAs) are another class of RNAs that can sponge microRNAs, thus regulating their functions in embryogenesis [243,244]. CircRNAs are single-stranded RNA molecules generated by back-splicing reactions [245]. A circRNA map of transcripts has been associated with naïve and primed pluripotency of hiPSCs generated from cord blood MSCs, and numerous studies also revealed that they regulate stem-cell differentiation [246,247]. *RMST* and *FIRRE* have been identified as lncRNAs, that are processed as circular lncRNAs during hESC differentiation (H9 cell line, WiCell) [248]. Similarly, the two circular RNAs, *circBIRC6* and *circCORO1C*, have been associated with the pluripotent state of hESCs (H9 cell line), where they interact with *miR-34a* and *miR-145* to modulate hESC pluripotency and differentiation [249]. CircRNAs are also involved in human cancer development (liver, lung, colorectal, breast, prostate, bladder, ovarian, kidney, and gastric cancers, hematological malignancies, and tumors of the central nervous system) and progression, and, to date, they are considered potential diagnostic and prognostic biomarkers [250]. MiOncoCirc is the first database containing circRNAs directly detected in tumor tissues, which allows identifying circRNA candidates as biomarkers for prostate cancer [251]. The circRNAs expressed in ESCs are often found overexpressed in cancer. For example, *RMST* is overexpressed in medulloblastoma, while *circBIRC6* promotes non-small-cell lung cancer cell progression by targeting *miR-145* and hepatocellular carcinoma by sponging *miR-3918* [252,253,254]. An oncogenic role has also been described for *circCORO1C*, which promotes the progression of laryngeal squamous cell carcinoma, competitively binding to *let-7c-5p* and inducing EMT [255]. CircRNAs are also aberrantly expressed in CSCs. In breast cancer stem cells, they could be involved in stemness inhibition, acting as miRNA sponges. In fact, the circRNA *VRK1* could inhibit the expansion and self-renewal ability of the breast CSCs, representing a good target for the therapeutic treatment of the tumor [256]. In CSC-enriched colorectal cancer spheroid cells, a circRNA–miRNA–mRNA axis has been identified and it is able to modulate stemness-related pathways. Two circRNAs (*hsa_circ_0066631* and *hsa_circ_0082096*), downregulating the expression of several microRNAs (*miR-140-3p*, *miR-224*, *miR-382*, *miR-548c-3p*, and *miR-579*), inhibit the degradation of transcripts involved in different pathways regulating CSC stemness [257]. In glioma stem cells, *circATP5*B competitively sponges *miR-185-5p*, upregulating the expression of the homeobox gene *HOXB5*, which induces the proliferation of glioma stem cells through JAK2/STAT3 signaling [258]. In the same context, the circRNA *cARF1* competitively binds to *miR-342-3p*, upregulating the expression of the transcription factor *ISL2*, which induces the expression of U2AF2 and causes an oncogenic effect [259]. In human liver cancer, the circRNA *CircMEG3* inhibits the growth of the liver cancer stem cells repressing the expression of the *m6A methyltransferase METTL3* and *Cbf5*, which in turn represses the telomerase activity [260].

Collectively, these data indicate that the mechanisms of post-transcriptional regulation can be highly complex and can involve different classes of RNAs, able to regulate each other.

## 8. Impacts of Tissue-Specific miRNAs on Adult Stem Cells and CSCs

The identity and function of certain tissues is guaranteed by the presence of microRNAs having tissue- or time-specific expression patterns [261]. Tissue-specific microRNAs (TS miRNAs) are a class of miRNAs expressed in specific tissues of our body. Their expression is regulated by non-tissue-specific transcription factors, whose binding sites are located near to the transcription start site of the TS miRNAs [262]. In our body, different miRNAs show a tissue-specific expression pattern. For example, *miR-122* is specifically expressed in the liver; *miR-9*, *miR-124*, and *miR- 128a/b* are specifically expressed in brain; *miR-7*, *miR-375*, *miR-141*, and *miR-200a* are specifically expressed in the pituitary gland and intestine; *miR-142* is specifically expressed in hematopoietic cells and the colon; *miR-1* is strongly expressed in human adult heart, with low levels in liver and midbrain; *miR-143* is particularly abundant in the spleen [263,264,265,266]. TS miRNAs have been also selectively identified in adult stem cells, as in the case of the mesenchymal stem cells (MSCs, Lonza) (*miR-196b*, *-196a*, *-615*, *-501*, *-449*, *-17-3p*, *-497*, and *-486*) and liver-resident stem cells, isolated from human cryopreserved normal adult hepatocytes (HLSCs, Lonza) (*miR-7*, *-95*, *-204*, and *-650*) [267]. Of interest, cell-derived microvesicles isolated from MSCs also contain patterns of miRNAs specifically expressed by MSCs (*miR-103-1*, *-140*, *-143-5p*, and *-340*), which are specific to cell origin and represent a peculiar signature for adult stem cells [267]. Moreover, the different expression of exosome-delivered microRNAs between somatic stem cells and CSCs also represents a good way to specifically identify the CSCs [268]. Indeed, the aberrant expression of microRNAs normally expressed in adult stem cells can be responsible for cancer development. In this section, we recapitulate the impacts of miRNAs on adult stem cells and CSCs, with particular attention paid to microRNAs differentially expressed between neural stem cells (NSCs) and brain tumor-initiating cells, intestinal stem cells (ISCs) and colon CSCs and mammary stem cells (MaSCs) and breast CSCs.

### 8.1. MiRNAs Expressed in Neural Stem Cells and Brain Tumor-Initiating Cells

NSCs are adult stem cells, located in the nervous system that, during embryogenesis, guarantee nervous tissue development, whereas, during adult life, they are reduced in number, remain quiescent, and are limited to specific areas of the brain [269]. Recent studies suggest that, in the adult tissue, NSCs are involved in neuronal plasticity, aging, disease, and regeneration [269]. MicroRNAs expressed in physiological condition in adult stem cells of the brain are often up- or downregulated in neural CSCs, causing tumor progression. For example, microRNAs expressed by neural stem cells such as *miR-7*, *-124*, *-125*, *-18, -9*, *-10*, and *-130*, orchestrating the differentiation of stem cells toward mature neuronal lineages during embryogenesis, are also expressed by neural cancer stem cells, where they exhibit tumor suppressor or oncogenic functions [270]. Indeed, microRNAs play crucial roles in brain tumor-initiating cells; for example, the aberrant upregulation of *miR-221* and the downregulation of the brain-enriched miRNAs *miR-128*, *miR-181a*, *miR-181b*, and *miR-181c* in the human glioblastoma cells lines (DBTRG-05MG, U118, U87, A172, LN18, M059J, M059K, LN229, T98G, and U138MG from ATCC) are responsible for tumor initiation and development [271,272]. Similarly, *miR-21* is upregulated in glioblastoma cell lines (A172, U87, U373, LN229, LN428, and LN308) compared with nonneoplastic glial cells and contributes to tumor malignancy by inhibiting the expression of apoptosis-related genes [273]. *MiR-340* is an additional example of microRNA expressed in human NSCs (H9, Invitrogen) and normal brain tissue, which is downregulated in glioma-initiating cells, contributing to proliferation and diffuse invasion of glioblastoma cells [274]. Forty-three microRNAs (including *miR-34a* and *miR-221/222* and novel miRNAs) have also been found deregulated in three separate CD133^+^ human glioblastoma cell lines compared to CD133^+^ normal NSCs [275]. This expression profile allowed distinguishing CSCs and NSCs that share the expression of the stem-cell marker CD133 [275]. The pro-neural microRNA *miR-218* has been also described as a tumor-suppressor miRNA, whose decreased expression correlates with the aggressiveness of glioma-initiating NSCs [276]. MicroRNAs are also differentially expressed between human glioblastoma CSCs and their paired autologous differentiated tumor cells, as in the case of *miR-21* and *miR-95,* which are significantly deregulated in glioblastoma CSCs [277].

### 8.2. MiRNAs Expressed in ISCs and Colon CSCs

ISCs are adult stem cells with roles in intestinal mucosa barrier homeostasis and repair, with self-renewal and differentiation ability [245]. The *Drosophila* adult intestine has been used as a model to detect TS miRNAs expressed in ISCs [278]. *MiR-958* is a TS miRNA, expressed in ISCs, that is transiently downregulated in stress conditions, causing an expansion of stem-cell number and controlling tissue regeneration [278]. Normal colon stem cells (Human T4056 cells, Applied Biological Materials Inc., Richmond, BC, Canada) also express high levels of *miR-137*, which is downregulated in colon CSCs (EpCAM⁺/CD44⁺/CD66a^−^, human SW480 cells, ATCC) [279]. In normal colon stem cells, this microRNA targets *doublecortin-like kinase 1 (DCLK1)* mRNA, and its stable expression suppresses their uncontrolled cell proliferation and tumorigenicity. In colon CSCs, the *DCLK1* transcript is highly expressed because of *miR-137* downregulation [279].

MicroRNAs in intestinal epithelial stem cells are also regulated by microbiota and chemoprotective dietary agents; perturbation in adult stem cells, caused by diet composition, is considered a trigger event for colon tumorigenesis [266,280]. *MiR-375* is a non-tissue-specific microRNA, significantly suppressed by the microbiota in ISCs, which appears to be an important regulator of stem-cell proliferation [266]. Of interest, *miR-375* knockdown increases the proliferation ability of the intestinal epithelial stem cells, and, although this microRNA is also expressed in other tissues, its sensitivity to the microbiota is a peculiar feature of ISCs [266]. In colorectal cancer cells (HT29, HCT116, and CaCO2), *miR-375* also functions as a tumor suppressor, targeting the JAK2/STAT3 and MAP3K8/ERK pathways [281].

### 8.3. MiRNAs Expressed in MaSCs and Breast CSCs

Adult MaSCs are multipotent stem cells, located in the mammary gland, with self-renewal and differentiation ability, responsible for tissue development, homeostasis, and expansion [282]. MaSCs express a unique miRNA signature, mainly featuring the expression of the primate-specific miRNA cluster (19q13.4) [283]. Of interest, the expression of *C19MC*, which harbors around 50 mature miRNAs, also represents a peculiar hallmark of triple-negative breast cancers; it is upregulated in tamoxifen-resistant cells, with *miRNA-519a* as the most highly upregulated miRNA [284,285]. *Mir-31* is another example of microRNAs highly expressed in MaSCs, which is also enriched in mammary tumors [286]. *Mir-31* is regulated by NF-kB signaling, and it promotes MaSC proliferation and expansion by modulating different pathways, such as Wnt/β-catenin [286]. Similarly, *miR-146a/b* is highly expressed in MaSCs and breast CSCs, where it reduces the adaptive response mechanism and promotes the exit from the quiescent state, inducing resistance to chemotherapies [287]. *MiR-489* is highly expressed in MaSCs and its overexpression inhibits mammary gland development, with a specific impact on cells with a high proliferation rate (CD49f^hi^ CD61^hi^ populations in the tumors), inhibiting tumor growth and metastasis [288]. MaSCs are also characterized by the high expression of *miR-205* and *miR-22* and by the downregulation of *let-7* and *miR-93* [289]. *MiR-205* is aberrantly expressed in breast cancer tissues, and a decrease in its expression correlates with the aggressiveness of the breast cancer phenotype; therefore, its downregulation is associated with poor prognosis and can be used as tumor biomarker [290]. Studies conducted in conditional mammary gland-specific transgenic mouse models revealed that *miR-22* promotes the expansion of the stem-cell compartment, inducing tumor development and aggressive metastatic disease [291]. Lastly, *miR-93* is also significantly downregulated in chemoresistant breast cancer cell lines (BCap37, Cell Bank of the Chinese Scientific Academy; Bats-72 and Bads-200 established by PTX treatment of parental BCap37 cells) and tumor samples, where it inhibits cell proliferation, inducing G1/S cell-cycle arrest and increasing the chemosensitivity to the pharmacological treatment [292].

## 9. Conclusions

Growing evidence reveals that the mechanisms governing the post-transcriptional regulation of gene expression can be highly complex, and they feature the action of different RNA molecules. The discovery of new classes of microRNAs strongly changed the old concept according to which these short noncoding RNA molecules could only regulate the translation of their direct mRNA targets. The functions mediated by the new classes of noncanonical miRNAs, together with their ability to act using noncanonical gene targeting, further complicate the process of gene expression regulation.

MicroRNAs orchestrate numerous functions in ESCs, such as pluripotency, self-renewal, differentiation, and EMT [4,35]. These processes are spatially and temporally regulated, so that the cells can correctly develop and guarantee a proper embryogenesis during development. During adult life, the lack of this fine regulation can be responsible for the dedifferentiation and deregulation of normal cells, causing their transformation into cancer cells and the acquisition of stem-like properties [175,176,177]. Collectively, the results discussed in this review highlight that microRNAs can orchestrate similar circuits in ESCs and cancer cells (Table 1). Another example in support of this emerging idea is represented by the *mir-23a-24-27a* cluster and *miR-125a/b* family. The *mir-23a-24-27a* cluster is activated in ESCs (E14Tg2a cell line, Bay Genomics), where it protects the cells against BMP4-induced apoptosis during differentiation [106]. This cluster also functions as an oncogene in several human cancers, such as human hepatocellular carcinoma and lung cancer, where it acts as an antiapoptotic and proliferation-promoting factor and reduces E-Cadherin expression to induce EMT [107,108]. *MiR-125a* and *miR-125b* target the BMP4-coreceptor *DIES1* in ESCs (E14Tg2a cell line, Bay Genomics), downregulating BMP4 signaling and promoting the exit of ESCs from the naïve state [109,110,111]. The suppression of these microRNAs is essential for the maintenance of the stem-cell properties of hepatoblasts and probably cancer cells, although this remains to be discovered [293]. *MiR-125a* and *miR-125b* could have a double role, because, in CSCs of hepatocellular carcinoma, they also inhibit cancer-associated macrophages, limiting tumor progression [112]. Importantly, the findings collected so far indicate that microRNAs, functioning as oncogenes or tumor suppressors, can be used as tumor biomarkers or therapeutic targets [294,295]. In some tumors, such as multiple myeloma, preliminary basic studies and associated clinical works have explored the value of microRNAs as potential biomarkers [296]. In lung cancer, oncolytic virotherapy and nanotherapy have been used to deliver microRNAs into cancer cells, although they have not been included in cancer treatments so far [297]. Several clinical studies are evaluating the utility of miRNA-blood based analyses for the early detection of cancer, while others are investigating the prognostic and predictive value of these molecules [298]. Currently, it is difficult to convert the results obtained from research studies into clinical trials, because microRNAs are widely expressed in different tissues and have wide-ranging effects, whereas some limitations are also linked to the technologies used for their delivery. Many years of hard work are still necessary to solve this gap.

## Figures and Tables

**Table 1:** MicroRNAs expressed in ESCs and CSCs and their relative functions.

miRNA	Functions in ESCs	ESC Cell Line	Source	Functions in CSCs	CSC Isolation
*miR-200*	Regulation of self-renewal and differentiation [47]. Regulation of EMT process by repression of *Zeb1* and *Snal1* TFs [48,49,50].	H9 and HUES6 cell lines H9 and H1 cell lines/MC50 and modified A2lox cells.	WiCell WiCell Institute/Dr. Robert Schreiber [51].	Repressed in human pancreatic cancer stem cells. Its overexpression inhibits EMT, thereby decreasing colony formation, chemoresistance, and invasion [52] Repressed in colon cancer cells and human colorectal cancer tissues by *NANOG* and *ZEB1* TFs, and sponged by *lncATB* to induce EMT, cell dissemination, and metastases [53,54,55,56].	Human pancreatic cancer stem cells were isolated from PANC-1 cell line by FACS [52].
*miR-451*	Upregulated during ESC differentiation toward erythroid lineage [57].	E14Tg2a cell line.	Not Reported.	Tumor-suppressor gene in most cancer types, such as early-stage breast cancer, follicular thyroid tumor, lung adenocarcinoma, and multiple myeloma. Its downregulation is used as biomarker for early cancer diagnosis [58,59,60,61].	
*miR-21*	Nuclear microRNA involved in the regulation of ESC pluripotency. It directly targets *Sox2*, decreasing its expression and reducing ESC self-renewal [62,63].	E14Tg2a.4 cell line.	Bay Genomics.	Highly expressed in cancer stem/progenitor cells isolated from ovarian teratocarcinoma PA1 cells, with a potential role to mediate growth and self-renewal of CSCs [64].	Isolated from human ovarian teratocarcinoma PA1 cells by FACS [65].
*miR-29*	It contributes to the early differentiation of ESCs, repressing the expression of *Tet1* transcript and promoting the upregulation of trophoblast lineage markers [66].	E14Tg2a cell line.	Not reported.	In ovarian cancer, it inhibits glycolysis and glucose metabolism, directly targeting *AKT2* and *AKT3* [67,68]. In melanoma cells, it mediates antiproliferative effects by downregulating CDK6, a regulator of G1/S phase [69].	
*miR-320*	Noncanonical miRNA that induces the proliferation of *Dcgr8*-deficient ESCs, downregulating the expression of the cell-cycle inhibitors p57 and p21 [70].	Germline-competent wild-type (W4), *Dgcr8*-deficient (*Dgcr8*^Δ/Δ^), and *Dicer*-deficient (Dicer^Δ/Δ^) cells.	[71]	Tumor-suppressor miRNA downregulated in breast cancer, glioma, gastric cancer, retinoblastoma, and human non-small-cell lung cancer. It is mainly involved in the inhibition of EMT, reducing the levels of E-Cadherin and increasing that of N-Cadherin and Vimentin [72,73,74,75,76,77,78].	
*miR-30*	Together with *let-7*, *mir-125*, and *mir-9*, it downregulates the expression of the RNA-binding protein LIN28, directly binding its 3′-UTR [79].	R1 and C57BL/6J-693 cell lines.	ATCC/The Jackson Laboratory.	The mechanism acting in ESCs is reversed in the LIN28-positive human breast cancer cell line, where LIN28 downregulates *miR-30*, *let-7*, *miR-125*, and *miR-9*. This could be responsible for LIN28 reactivation in a cancer context [79].	
*miR-122*	Regulator of ESC differentiation, acting in the *miR-122*/*FoxA1*/HNF4a-positive feedback loop. Its overexpression promotes the hepatic differentiation of ESCs [80].	Mouse ESCs (no details reported).	Cyagen Company.	MicroRNA expressed at low levels in hepatocellular carcinoma. Its expression inversely correlates with the levels of the G9A histone methyltransferase, causing worst poor overall survival of patients [81].	
*miR-155*	Expressed in ESCs and gradually downregulated during ESC cardiac differentiation. Its inhibition promotes cardiogenesis, involving RAS-ERK1/2 signaling and the myogenic enhance factor 2C [82].	CGR8 cell line.	ECACC.	Oncogenic miRNA overexpressed in different cancer cells with stem-like properties, such as breast cancer. Its inhibition significantly reduces the proliferation of invasive breast cancer cell lines, while its overexpression promotes the acquisition of stem-like properties [83].	
*miR-302*	Involved in pluripotency maintenance of ESCs, through the downregulation of the inhibitors of G1/S cell-cycle transition [84]. OCT4 and SOX2 bind a conserved region in its promoter, such that its expression follows *OCT4* expression during embryogenesis [85].	*Dgcr8* knockout (*Dgcr8*^Δ/Δ^) ESCs. H1 and BG-01 cell lines, hESBGN-01.	[86] WiCell. Glioma-initiating primary cell lines TG1, TG6, GB1, isolated from human glioblastoma.	In P19 mouse embryonic carcinoma cells, it is transcriptionally activated by OCT4 [87]. Pluripotency inducer. It represses the transcripts involved in differentiation processes and maintains high levels of the pluripotency factor OCT4 [88]. Prevention of human induced pluripotent stem-cell tumorigenicity by reduction of the G1–S cell-cycle transition (suppression of Cyclin E-CDK2 and Cyclin D-CDK4/6 expression) and induction of the senescence-associated tumor-suppressor genes [89]. Pluripotency repressor in glioblastoma-initiating cancer cells. Its ectopic expression represses OCT4 and NANOG. The inhibition of the stemness signatures and tumorigenic properties of glioma-initiating cancer cells is mediated by the drastic downregulation of CXCR4/SDF1 pathway and inhibition of the expression of the cell-cycle-related transcripts *E2F1*, *cyclinA*, and *cyclin D* [90].	
*let-7 miRNAs*	Involved in differentiation of ESCs [37]. The expression of miRNAs belonging to *let-7* family is inhibited by LIN28 protein, to maintain the cells in their undifferentiated state [91].	*Dgcr8*^−/−^ and wild-type V6.5 ESCs.	[86]	Well-known tumor-suppressor miRNA, involved in different cancers such as non-small-cell lung cancer, breast cancer, and multiple myeloma; its downregulation contributes to carcinogenesis, increasing the stemness factors. In rare cases (i.e., lung cancer cells, oral cavity squamous cell carcinomas), it can act as an oncogene, increasing cancer migration, invasion, and progression [92,93,94,95,96,97].	
*C19MC miRNA cluster*	Expressed in placenta and ESCs. Its activation is responsible for suppression of EMT-related genes and induction of OCT4 and FGF4 expression [98].	HTR8/SVneo cells.	ATCC.	Transcriptional hallmark of different types of cancers (type A and AB thymomas, hepatocellular carcinoma, undifferentiated embryonal sarcoma of the liver, embryonal tumor with multilayered rosettes, etc.). miRNA cluster frequently affected by chromosomal rearrangements [99,100,101,102,103].	
*miR-429*	EMT suppressor acting during embryo implantation, through the targeting of members belonging to Cadherins family [104].	C57BL6/J and BALB/C mice.	[104]	Tumor suppressor in colon cancer, thanks to the direct binding to *HMGB3* oncogene [105].	
*Mir-23a-24-27a* cluster	Activated in ESCs to protect the cells against BMP4-induced apoptosis during differentiation [106].	E14Tg2a cell line.	Bay Genomics.	Oncogenic cluster involved in different human cancers (hepatocellular carcinoma, lung cancer, etc.), where it acts as an antiapoptotic, proliferation, and EMT-promoting factor [107,108].	
*MiR-125a/b family*	Inducer of ESC exit from the naïve state by binding the BMP4 coreceptor *DIES1* [109,110,111].	E14Tg2a cell line.	Bay Genomics.	In CSCs of hepatocellular carcinoma, they inhibit cancer-associated macrophages, limiting tumor progression [112].	

**Table 2:** Noncanonical miRNAs acting in ESCs and CSCs.

**Type of Noncanonical miRNA**	**MiRNA Name**	**Impact in ESCs**
Mirtron	*miR-novel-41*	Predominantly expressed in mouse ESCs. It is derived from the intron of the *Man2c1* gene and is conserved between mouse and rat [114].
	*mir-702*	Expressed in mouse ESCs. *MiR-702* promotes the proliferation of *Dgcr8*-deficient ESCs, unlocking the arrest in G1 phase of the cell cycle [70,115].
	*mir-877*	Expressed in mouse ESCs, where its mirtronic identity has been confirmed. It is conserved across mouse, humans, and chimps [115].
	*mir-1981*	Expressed in mouse ESCs [115].
	*mir-1224*	Expressed in mouse ESCs [116].
	*mir-3082*	Expressed in mouse ESCs [116].
	*mir-3102*	Expressed in mouse ESCs [116].
Small nucleolar RNAs	*SNORD12, 29, 31, 74, 101, and 104*	Abundantly expressed in ESCs (E14Tg2a). Their expression is downregulated upon ESC differentiation [117].
	*H/ACA* *snoRNAs*	H/ACA snoRNA families are differentially expressed during the differentiation of mouse ESCs (E14Tg2a) with retinoic acid. Some of them are abundantly expressed in mESCs, while others are highly expressed in retinoic acid-differentiated cells [118].
**Type of Noncanonical miRNA**	**MiRNA Name**	**Impact in CSCs and Cancer Cells**
Mirtron	*miR-6778-5*	5’-tail mirtron type that acts as critical regulator for maintenance of CSC stemness in *Drosha*-silenced gastric cancer cells [119].
	*miR-140*	*MiR-140* regulates CSCs in luminal subtype invasive ductal carcinoma. Downregulated in CSC-like cells. It targets the stem-cell factors *SOX9* and *ALDH1* in ductal carcinoma, regulating CSC self-renewal and tumor formation in vivo [120].
	*miR-1227-3p, miR-1229-3p, and miR-1236-3p*	*MiR-1229-3p* is upregulated in pancreatic (SU.86.86, T3M4) and stomach (KATOIII) cancer cell lines derived from metastatic sites. *MiR-1226-3p* is significantly expressed in stomach tumors and downregulated in colorectal tumors [121].
Small nucleolar RNAs	*SNORA80E, SNORA73B, SNORD33, SNORD66, SNORD76, and SNORD78*	Highly expressed in lung cancer tissues. *SNORA80E* knockdown in non-small-cell lung cancer cell lines (H460 and H1944) inhibits cell proliferation; it is also overexpressed in colorectal cancer [122].
	*SNORD89*	Highly expressed in ovarian cancer cells (OVCAR-3 (OV) and CAOV-3 (CA), ATCC), where it increases the expression of the stemness markers, cell proliferation, invasion, and migration [123]
	*SNORA21*	Overexpressed in colorectal adenomas and cancer. Its inhibition in SW48 cells decreases cell proliferation and invasion, modulating cancer-related pathways [124].

## References

[1] Young R.A. (2011). Control of the embryonic stem cell state. Cell.

[2] Evans M.J., Kaufman M.H. (1981). Establishment in culture of pluripotential cells from mouse embryos. Nature.

[3] Ying Q.L., Wray J., Nichols J., Batlle-Morera L., Doble B., Woodgett J., Cohen P., Smith A. (2008). The ground state of embryonic stem cell self-renewal. Nature.

[4] Divisato G., Passaro F., Russo T., Parisi S. (2020). The Key Role of MicroRNAs in Self-Renewal and Differentiation of Embryonic Stem Cells. Int. J. Mol. Sci..

[5] Gökbuget D., Blelloch R. (2019). Epigenetic control of transcriptional regulation in pluripotency and early differentiation. Development.

[6] Navarra A., Musto A., Gargiulo A., Petrosino G., Pierantoni G.M., Fusco A., Russo T., Parisi S. (2016). Hmga2 is necessary for Otx2-dependent exit of embryonic stem cells from the pluripotent ground state. BMC Biol..

[7] Passaro F., De Martino I., Zambelli F., Di Benedetto G., Barbato M., D’Erchia A.M., Manzari C., Pesole G., Mutarelli M., Cacchiarelli D. (2020). YAP contributes to DNA methylation remodeling upon mouse embryonic stem cell differentiation. J. Biol. Chem..

[8] Zimmerlin L., Park T.S., Zambidis E.T. (2017). Capturing Human Naïve Pluripotency in the Embryo and in the Dish. Stem. Cells Dev..

[9] Prochazkova M., Chavez M.G., Prochazka J., Felfy H., Mushegyan V., Klein O.D. (2015). Chapter 18—Embryonic Versus Adult Stem Cells. In Stem Cell Biology and Tissue Engineering in Dental Sciences.

[10] Islam F., Gopalan V., Lam A.K.Y. (2019). Chapter 6—Cancer Stem Cells: Role in Tumor Progression and Treatment Resistance. Oncogenomics.

[11] Virolle T. (2017). Cellules souches cancéreuses dans les glioblastomes [Cancer stem cells in glioblastoma]. Bull. Cancer.

[12] Chen K., Huang Y.H., Chen J.L. (2013). Understanding and targeting cancer stem cells: Therapeutic implications and challenges. Acta Pharmacol. Sin..

[13] Clarke M.F., Dick J.E., Dirks P.B., Eaves C.J., Jamieson C.H., Jones D.L., Visvader J., Weissman I.L., Wahl G.M. (2006). Cancer stem cells--perspectives on current status and future directions: AACR Workshop on cancer stem cells. Cancer Res..

[14] Tomasetti C., Vogelstein B. (2015). Variation in cancer risk among tissues can be explained by the number of stem cell divisions. Science.

[15] Yu Z., Pestell T.G., Lisanti M.P., Pestell R.G. (2012). Cancer stem cells. Int. J. Biochem. Cell Biol..

[16] Reya T., Morrison S.J., Clarke M.F., Weissman I.L. (2001). Stem cells, cancer, and cancer stem cells. Natuure.

[17] Treisman D., Li Y., Zhu Y. (2021). Stem-like Cell Populations, p53-Pathway Activation and Mechanisms of Recurrence in Sonic Hedgehog Medulloblastoma. NeuroMolecular Med..

[18] Grassi E.S., Ghiandai V., Persani L. (2021). Thyroid Cancer Stem-like Cells: From Microenvironmental Niches to Therapeutic Strategies. J. Clin. Med..

[19] Zhou D., Luo Y., Dingli D., Traulsen A. (2019). The invasion of de-differentiating cancer cells into hierarchical tissues. PLoS Comput. Biol..

[20] Friedmann-Morvinski D., Verma I.M. (2014). Dedifferentiation and reprogramming: Origins of cancer stem cells. EMBO Rep..

[21] Carvalho J. (2020). Cell Reversal From a Differentiated to a Stem-like State at Cancer Initiation. Front. Oncol..

[22] Lu J., Ye X., Fan F., Xia L., Bhattacharya R., Bellister S., Tozzi F., Sceusi E., Zhou Y., Tachibana I. (2013). Endothelial cells promote the colorectal cancer stem cell phenotype through a soluble form of Jagged-1. Cancer Cell.

[23] Heddleston J.M., Li Z., McLendon R.E., Hjelmeland A.B., Rich J.N. (2009). The hypoxic microenvironment maintains glioblastoma stem cells and promotes reprogramming towards a cancer stem cell phenotype. Cell Cycle.

[24] Pardal R., Clarke M.F., Morrison S.J. (2003). Applying the principles of stem-cell biology to cancer. Nat. Rev. Cancer.

[25] Sell S., Pierce G.B. (1994). Maturation arrest of stem cell differentiation is a common pathway for the cellular origin of teratocarcinomas and epithelial cancers. Lab. Invest..

[26] Smith A. (2017). Formative pluripotency: The executive phase in a developmental continuum. Development.

[27] Pan G., Thomson J.A. (2007). Nanog and transcriptional networks in embryonic stem cell pluripotency. Cell Res..

[28] Nichols J., Zevnik B., Anastassiadis K., Niwa H., Klewe-Nebenius D., Chambers I., Schöler H., Smith A. (1998). Formation of pluripotent stem cells in the mammalian embryo depends on the POU transcription factor Oct4. Cell.

[29] Masui S., Nakatake Y., Toyooka Y., Shimosato D., Yagi R., Takahashi K., Okochi H., Okuda A., Matoba R., Sharov A.A. (2007). Pluripotency governed by Sox2 via regulation of Oct3/4 expression in mouse embryonic stem cells. Nat. Cell Biol..

[30] Luo W., Li S., Peng B., Ye Y., Deng X., Yao K. (2013). Embryonic Stem Cells Markers SOX2, OCT4 and Nanog Expression and Their Correlations with Epithelial-Mesenchymal Transition in Nasopharyngeal Carcinoma. PLoS ONE.

[31] Zhang R., Xia J., Wang Y., Cao M., Jin D., Xue W., Huang Y., Chen H. (2020). Co-Expression of Stem Cell and Epithelial Mesenchymal Transition Markers in Circulating Tumor Cells of Bladder Cancer Patients. OncoTargets Ther..

[32] Yang L., Shi P., Zhao G., Xu J., Peng W., Zhang J., Zhang G., Wang X., Dong Z., Chen F. (2020). Targeting cancer stem cell pathways for cancer therapy. Signal Transduct. Target. Ther..

[33] Hüser L., Novak D., Umansky V., Altevogt P., Utikal J. (2018). Targeting SOX2 in anticancer therapy. Expert Opin. Ther. Targets.

[34] Gangemi R.M., Griffero F., Marubbi D., Perera M., Capra M.C., Malatesta P., Ravetti G.L., Zona G.L., Daga A., Corte G. (2009). SOX2 silencing in glioblastoma tumor-initiating cells causes stop of proliferation and loss of tumorigenicity. Stem Cells.

[35] Navarro A., Monzo M. (2010). MicroRNAs in human embryonic and cancer stem cells. Yonsei Med J..

[36] Bartel D.P. (2009). MicroRNAs: Target recognition and regulatory functions. Cell.

[37] Melton C., Judson R.L., Blelloch R. (2010). Opposing microRNA families regulate self-renewal in mouse embryonic stem cells. Nature.

[38] Chen Y., Liersch R., Detmar M. (2012). The miR-290-295 cluster suppresses autophagic cell death of melanoma cells. Sci. Rep..

[39] Fareh M., Almairac F., Turchi L., Burel-Vandenbos F., Paquis P., Fontaine D., Lacas-Gervais S., Junier M.P., Chneiweiss H., Virolle T. (2017). Cell-based therapy using miR-302-367 expressing cells represses glioblastoma growth. Cell Death Dis..

[40] Guo Y., Cui J., Ji Z., Cheng C., Zhang K., Zhang C., Chu M., Zhao Q., Yu Z., Zhang Y. (2017). miR-302/367/LATS2/YAP pathway is essential for prostate tumor-propagating cells and promotes the development of castration resistance. Oncogene.

[41] Viswanathan S.R., Daley G.Q., Gregory R.I. (2008). Selective blockade of microRNA processing by Lin28. Science.

[42] Parisi S., Passaro F., Russo L., Musto A., Navarra A., Romano S., Petrosino G., Russo T. (2017). Lin28 is induced in primed embryonic stem cells and regulates let-7-independent events. FASEB J..

[43] Balzeau J., Menezes M.R., Cao S., Hagan J.P. (2017). The LIN28/let-7 Pathway in Cancer. Front. Genet..

[44] Denli A.M., Tops B.B., Plasterk R.H., Ketting R.F., Hannon G.J. (2004). Processing of primary microRNAs by the microprocessor complex. Nature.

[45] Hutvágner G., McLachlan J., Pasquinelli A.E., Bálint E., Tuschl T., Zamore P.D. (2001). A cellular function for the RNA interference enzyme Dicer in the maturation of the let-7 small temporal RNA. Science.

[46] Hammond S.M., Boettcher S., Caudy A.A., Kobayashi R., Hannon G.J. (2001). Argonaute2, a link between genetic and biochemical analyses of RNAi. Science.

[47] Huang H.N., Chen S.Y., Hwang S.M., Yu C.C., Su M.W., Mai W., Wang H.W., Cheng W.C., Schuyler S.C., Ma N. (2014). miR-200c and GATA binding protein 4 regulate human embryonic stem cell renewal and differentiation. Stem Cell Res..

[48] Du Z.W., Ma L.X., Phillips C., Zhang S.C. (2013). miR-200 and miR-96 families repress neural induction from human embryonic stem cells. Development.

[49] Gill J.G., Langer E.M., Lindsley R.C., Cai M., Murphy T.L., Kyba M., Murphy K.M. (2011). Snail and the microRNA-200 family act in opposition to regulate epithelial-to-mesenchymal transition and germ layer fate restriction in differentiating ESCs. Stem Cells.

[50] Kim D.H., Xing T., Yang Z., Dudek R., Lu Q., Chen Y.H. (2017). Epithelial Mesenchymal Transition in Embryonic Development, Tissue Repair and Cancer: A Comprehensive Overview. J. Clin. Med..

[51] Iacovino M., Hernandez C., Xu Z., Bajwa G., Prather M., Kyba M. (2009). A conserved role for Hox paralog group 4 in regulation of hematopoietic progenitors. Stem Cells Dev..

[52] Ma C., Ding Y.C., Yu W., Wang Q., Meng B., Huang T. (2015). MicroRNA-200c overexpression plays an inhibitory role in human pancreatic cancer stem cells by regulating epithelial-mesenchymal transition. Minerva Med..

[53] Pan Q., Meng L., Ye J., Wei X., Shang Y., Tian Y., He Y., Peng Z., Chen L., Chen W. (2017). Transcriptional repression of miR-200 family members by Nanog in colon cancer cells induces epithelial-mesenchymal transition (EMT). Cancer Lett..

[54] Wellner U., Schubert J., Burk U.C., Schmalhofer O., Zhu F., Sonntag A., Waldvogel B., Vannier C., Darling D., zur Hausen A. (2009). The EMT-activator ZEB1 promotes tumorigenicity by repressing stemness-inhibiting microRNAs. Nat. Cell Biol..

[55] Title A.C., Hong S.J., Pires N.D., Hasenöhrl L., Godbersen S., Stokar-Regenscheit N., Bartel D.P., Stoffel M. (2018). Genetic dissection of the miR-200-Zeb1 axis reveals its importance in tumor differentiation and invasion. Nat. Commun..

[56] Li R.H., Chen M., Liu J., Shao C.C., Guo C.P., Wei X.L., Li Y.C., Huang W.H., Zhang G.J. (2018). Long noncoding RNA ATB promotes the epithelial-mesenchymal transition by upregulating the miR-200c/Twist1 axe and predicts poor prognosis in breast cancer. Cell Death Dis..

[57] Shokri G., Kouhkan F., Nojehdehi S., Soleimani M., Pourfathollah A.A., Zarif M.N., Tamaddon M., Obeidi N. (2019). Simultaneous regulation of miR-451 and miR-191 led to erythroid fate decision of mouse embryonic stem cell. Iran. J. Basic Med. Sci..

[58] Bai H., Wu S. (2019). miR-451: A Novel Biomarker and Potential Therapeutic Target for Cancer. OncoTargets Ther..

[59] Itani M.M., Nassar F.J., Tfayli A.H., Talhouk R.S., Chamandi G.K., Itani A.R.S., Makoukji J., Boustany R.N., Hou L., Zgheib N.K. (2021). A Signature of Four Circulating microRNAs as Potential Biomarkers for Diagnosing Early-Stage Breast Cancer. Int J. Mol. Sci..

[60] Knyazeva M., Korobkina E., Karizky A., Sorokin M., Buzdin A., Vorobyev S., Malek A. (2020). Reciprocal Dysregulation of MiR-146b and MiR-451 Contributes in Malignant Phenotype of Follicular Thyroid Tumor. Int. J. Mol. Sci..

[61] Huang J., Pan B., Xia G., Zhu J., Li C., Feng J. (2020). LncRNA SNHG15 regulates EGFR-TKI acquired resistance in lung adenocarcinoma through sponging miR-451 to upregulate MDR-1. Cell Death Dis..

[62] Meister G., Landthaler M., Patkaniowska A., Dorsett Y., Teng G., Tuschl T. (2004). Human Argonaute2 mediates RNA cleavage targeted by miRNAs and siRNAs. Mol. Cell.

[63] Singh S.K., Marisetty A., Sathyan P., Kagalwala M., Zhao Z., Majumder S. (2015). REST-miR-21-SOX2 axis maintains pluripotency in E14Tg2a.4 embryonic stem cells. Stem Cell Res..

[64] Kang H.Y. (2013). MicroRNA-21 regulates stemness in cancer cells. Stem Cell Res. Ther..

[65] Chung W.M., Chang W.C., Chen L., Chang Y.Y., Shyr C.R., Hung Y.C., Ma W.L. (2013). MicroRNA-21 promotes the ovarian teratocarcinoma PA1 cell line by sustaining cancer stem/progenitor populations in vitro. Stem Cell Res. Ther..

[66] Cui Y., Li T., Yang D., Li S., Le W. (2016). miR-29 regulates Tet1 expression and contributes to early differentiation of mouse ESCs. Oncotarget.

[67] Kwon J.J., Factora T.D., Dey S., Kota J. (2018). A Systematic Review of miR-29 in Cancer. Mol. Ther. Oncolytics.

[68] Teng Y., Zhang Y., Qu K., Yang X., Fu J., Chen W., Li X. (2015). MicroRNA-29B (mir-29b) regulates the Warburg effect in ovarian cancer by targeting AKT2 and AKT3. Oncotarget.

[69] Schmitt M.J., Philippidou D., Reinsbach S.E., Margue C., Wienecke-Baldacchino A., Nashan D., Behrmann I., Kreis S. (2012). Interferon-γ-induced activation of Signal Transducer and Activator of Transcription 1 (STAT1) up-regulates the tumor suppressing microRNA-29 family in melanoma cells. Cell Commun. Signal..

[70] Kim B.M., Choi M.Y. (2012). Non-canonical microRNAs miR-320 and miR-702 promote proliferation in Dgcr8-deficient embryonic stem cells. Biochem. Biophys. Res. Commun..

[71] Kanellopoulou C., Muljo S.A., Kung A.L., Ganesan S., Drapkin R., Jenuwein T., Livingston D.M., Rajewsky K. (2005). Dicer-deficient mouse embryonic stem cells are defective in differentiation and centromeric silencing. Genes Dev..

[72] Liang Y., Li S., Tang L. (2021). MicroRNA 320, an Anti-Oncogene Target miRNA for Cancer Therapy. Biomedicines.

[73] Zhang Z., Zhang J., Li J., Geng H., Zhou B., Zhang B., Chen H. (2020). miR-320/ELF3 axis inhibits the progression of breast cancer via the PI3K/AKT pathway. Oncol. Lett..

[74] Pan C., Gao H., Zheng N., Gao Q., Si Y., Zhao Y. (2017). MiR-320 inhibits the growth of glioma cells through downregulating PBX3. Biol. Res..

[75] Li B., Zhang H. (2017). Plasma microRNA-320 is a potential diagnostic and prognostic bio-marker in gastric cancer. Int J. Clin. Exp. Pathol..

[76] Zhao Y., Zhang S., Zhang Y. (2017). MicroRNA-320 inhibits cell proliferation, migration and invasion in retinoblastoma by targeting specificity protein 1. Mol. Med. Rep..

[77] Lei T., Zhu Y., Jiang C., Wang Y., Fu J., Fan Z., Qin H. (2016). MicroRNA-320 was downregulated in non-small cell lung cancer and inhibited cell proliferation, migration and invasion by targeting fatty acid synthase. Mol. Med. Rep..

[78] Hong H., Zhu H., Zhao S., Wang K., Zhang N., Tian Y., Li Y., Wang Y., Lv X., Wei T. (2019). The novel circCLK3/miR-320a/FoxM1 axis promotes cervical cancer progression. Cell Death Dis..

[79] Zhong X., Li N., Liang S., Huang Q., Coukos G., Zhang L. (2010). Identification of microRNAs regulating reprogramming factor LIN28 in embryonic stem cells and cancer cells. J. Biol. Chem..

[80] Deng X.G., Qiu R.L., Wu Y.H., Li Z.X., Xie P., Zhang J., Zhou J.J., Zeng L.X., Tang J., Maharjan A. (2014). Overexpression of miR-122 promotes the hepatic differentiation and maturation of mouse ESCs through a miR-122/FoxA1/HNF4a-positive feedback loop. Liver Int..

[81] Yuan L.T., Lee W.J., Yang Y.C., Chen B.R., Yang C.Y., Chen M.W., Chen J.Q., Hsiao M., Chien M.H., Hua K.T. (2021). Histone Methyltransferase G9a-Promoted Progression of Hepatocellular Carcinoma Is Targeted by Liver-Specific Hsa-miR-122. Cancers.

[82] Ling X., Yao D., Kang L., Zhou J., Zhou Y., Dong H., Zhang K., Zhang L., Chen H. (2017). Involment of RAS/ERK1/2 signaling and MEF2C in miR-155-3p inhibition-triggered cardiomyocyte differentiation of embryonic stem cell. Oncotarget.

[83] Zuo J., Yu Y., Zhu M., Jing W., Yu M., Chai H., Liang C., Tu J. (2018). Inhibition of miR-155, a therapeutic target for breast cancer, prevented in cancer stem cell formation. Cancer Biomark..

[84] Wang Y., Baskerville S., Shenoy A., Babiarz J.E., Baehner L., Blelloch R. (2008). Embryonic stem cell-specific microRNAs regulate the G1-S transition and promote rapid proliferation. Nat. Genet..

[85] Card D.A., Hebbar P.B., Li L., Trotter K.W., Komatsu Y., Mishina Y., Archer T.K. (2008). Oct4/Sox2-regulated miR-302 targets cyclin D1 in human embryonic stem cells. Mol. Cell Biol..

[86] Wang Y., Medvid R., Melton C., Jaenisch R., Blelloch R. (2007). DGCR8 is essential for microRNA biogenesis and silencing of embryonic stem cell self-renewal. Nat. Genet..

[87] Liu H., Deng S., Zhao Z., Zhang H., Xiao J., Song W., Gao F., Guan Y. (2011). Oct4 regulates the miR-302 cluster in P19 mouse embryonic carcinoma cells. Mol. Biol. Rep..

[88] Li H.L., Wei J.F., Fan L.Y., Wang S.H., Zhu L., Li T.P., Lin G., Sun Y., Sun Z.J., Ding J. (2016). miR-302 regulates pluripotency, teratoma formation and differentiation in stem cells via an AKT1/OCT4-dependent manner. Cell Death Dis..

[89] Lin S.L., Chen J.S., Ying S.Y. (2020). MiR-302-Mediated Somatic Cell Reprogramming and Method for Generating Tumor-Free iPS Cells Using miR-302. Methods Mol. Biol..

[90] Fareh M., Turchi L., Virolle V., Debruyne D., Almairac F., de-la-Forest Divonne S., Paquis P., Preynat-Seauve O., Krause K.H., Chneiweiss H. (2012). The miR 302-367 cluster drastically affects self-renewal and infiltration properties of glioma-initiating cells through CXCR4 repression and consequent disruption of the SHH-GLI-NANOG network. Cell Death Differ..

[91] Piskounova E., Polytarchou C., Thornton J.E., LaPierre R.J., Pothoulakis C., Hagan J.P., Iliopoulos D., Gregory R.I. (2011). Lin28A and Lin28B inhibit let-7 microRNA biogenesis by distinct mechanisms. Cell.

[92] Chirshev E., Oberg K.C., Ioffe Y.J., Unternaehrer J.J. (2019). Let-7 as biomarker, prognostic indicator, and therapy for precision medicine in cancer. Clin. Transl. Med..

[93] Dai X., Fan W., Wang Y., Huang L., Jiang Y., Shi L., Mckinley D., Tan W., Tan C. (2016). Combined Delivery of Let-7b MicroRNA and Paclitaxel via Biodegradable Nanoassemblies for the Treatment of KRAS Mutant Cancer. Mol. Pharm..

[94] Peng F., Li T.T., Wang K.L., Xiao G.Q., Wang J.H., Zhao H.D., Kang Z.J., Fan W.J., Zhu L.L., Li M. (2017). H19/let-7/LIN28 reciprocal negative regulatory circuit promotes breast cancer stem cell maintenance. Cell Death Dis..

[95] Manier S., Powers J.T., Sacco A., Glavey S.V., Huynh D., Reagan M.R., Salem K.Z., Moschetta M., Shi J., Mishima Y. (2017). The LIN28B/let-7 axis is a novel therapeutic pathway in multiple myeloma. Leukemia.

[96] Brueckner B., Stresemann C., Kuner R., Mund C., Musch T., Meister M., Sültmann H., Lyko F. (2007). The human let-7a-3 locus contains an epigenetically regulated microRNA gene with oncogenic function. Cancer Res..

[97] Hilly O., Pillar N., Stern S., Strenov Y., Bachar G., Shomron N., Shpitzer T. (2016). Distinctive pattern of let-7 family microRNAs in aggressive carcinoma of the oral tongue in young patients. Oncol. Lett..

[98] Mong E.F., Yang Y., Akat K.M., Canfield J., VanWye J., Lockhart J., Tsibris J.C.M., Schatz F., Lockwood C.J., Tuschl T. (2020). Chromosome 19 microRNA cluster enhances cell reprogramming by inhibiting epithelial-to-mesenchymal transition. Sci. Rep..

[99] Radovich M., Solzak J.P., Hancock B.A., Conces M.L., Atale R., Porter R.F., Zhu J., Glasscock J., Kesler K.A., Badve S.S. (2016). A large microRNA cluster on chromosome 19 is a transcriptional hallmark of WHO type A and AB thymomas. Br. J. Cancer.

[100] Rui T., Xu S., Zhang X., Huang H., Feng S., Zhan S., Xie H., Zhou L., Ling Q., Zheng S. (2020). The chromosome 19 microRNA cluster, regulated by promoter hypomethylation, is associated with tumour burden and poor prognosis in patients with hepatocellular carcinoma. J. Cell Physiol..

[101] Setty B.A., Jinesh G.G., Arnold M., Pettersson F., Cheng C.H., Cen L., Yoder S.J., Teer J.K., Flores E.R., Reed D.R. (2020). The genomic landscape of undifferentiated embryonal sarcoma of the liver is typified by C19MC structural rearrangement and overexpression combined with TP53 mutation or loss. PLoS Genet..

[102] Jinesh G.G., Napoli M., Smallin M.T., Davis A., Ackerman H.D., Raulji P., Montey N., Flores E.R., Brohl A.S. (2021). Mutant p53s and chromosome 19 microRNA cluster overexpression regulate cancer testis antigen expression and cellular transformation in hepatocellular carcinoma. Sci. Rep..

[103] Mayr L., Gojo J., Peyrl A., Azizi A.A., Stepien N.M., Pletschko T., Czech T., Dorfer C., Lambo S., Dieckmann K. (2020). Potential Importance of Early Focal Radiotherapy Following Gross Total Resection for Long-Term Survival in Children With Embryonal Tumors With Multilayered Rosettes. Front. Oncol..

[104] Li Z., Gou J., Jia J., Zhao X. (2015). MicroRNA-429 functions as a regulator of epithelial-mesenchymal transition by targeting Pcdh8 during murine embryo implantation. Hum. Reprod..

[105] Tian X., Chang J., Zhang N., Wu S., Liu H., Yu J. (2021). MicroRNA-429 acts as a tumor suppressor in colorectal cancer by targeting high mobility group box 3. Oncol. Lett..

[106] Musto A., Navarra A., Vocca A., Gargiulo A., Minopoli G., Romano S., Romano M.F., Russo T., Parisi S. (2015). miR-23a, miR-24 and miR-27a Protect Differentiating ESCs From BMP4-induced Apoptosis. Cell Death Differ..

[107] Huang S., He X., Ding J., Liang L., Zhao Y., Zhang Z., Yao X., Pan Z., Zhang P., Li J. (2008). Upregulation of miR-23a approximately 27a approximately 24 decreases transforming growth factor-beta-induced tumor-suppressive activities in human hepatocellular carcinoma cells. Int J. Cancer.

[108] Cao M., Seike M., Soeno C., Mizutani H., Kitamura K., Minegishi Y., Noro R., Yoshimura A., Cai L., Gemma A. (2012). MiR-23a regulates TGF-β-induced epithelial-mesenchymal transition by targeting E-cadherin in lung cancer cells. Int J. Oncol..

[109] Parisi S., Battista M., Musto A., Navarra A., Tarantino C., Russo T. (2012). A Regulatory Loop Involving Dies1 and miR-125a Controls BMP4 Signaling in Mouse Embryonic Stem Cells. FASEB J..

[110] Battista M., Musto A., Navarra A., Minopoli G., Russo T., Parisi S. (2013). miR-125b Regulates the Early Steps of ESC Differentiation Through dies1 in a TGF-independent Manner. Int. J. Mol. Sci..

[111] Aloia L., Parisi S., Fusco L., Pastore L., Russo T. (2010). Differentiation of Embryonic Stem Cells 1 (Dies1) Is a Component of Bone Morphogenetic Protein 4 (BMP4) Signaling Pathway Required for Proper Differentiation of Mouse Embryonic Stem Cells. J. Biol. Chem..

[112] Wang Y., Wang B., Xiao S., Li Y., Chen Q. (2019). miR-125a/b inhibits tumor-associated macrophages mediated in cancer stem cells of hepatocellular carcinoma by targeting CD90. J. Cell Biochem..

[113] Breving K., Esquela-Kerscher A. (2010). The complexities of microRNA regulation: Mirandering around the rules. Int. J. Biochem. Cell Biol..

[114] Zhao B., Fan C., Jin Y. (2020). Identification of novel miRNAs in mouse embryonic stem cells and reprogrammed pluripotent cells. Acta Biochim. Biophys. Sin..

[115] Babiarz J.E., Ruby J.G., Wang Y., Bartel D.P., Blelloch R. (2008). Mouse ES cells express endogenous shRNAs, siRNAs, and other Microprocessor-independent, Dicer-dependent small RNAs. Genes Dev..

[116] Jouneau A., Ciaudo C., Sismeiro O., Brochard V., Jouneau L., Vandormael-Pournin S., Coppée J.Y., Zhou Q., Heard E., Antoniewski C. (2012). Naive and primed murine pluripotent stem cells have distinct miRNA expression profiles. RNA.

[117] Skreka K., Schafferer S., Nat I.R., Zywicki M., Salti A., Apostolova G., Griehl M., Rederstorff M., Dechant G., Hüttenhofer A. (2012). Identification of differentially expressed non-coding RNAs in embryonic stem cell neural differentiation. Nucleic Acids Res..

[118] McCann K.L., Kavari S.L., Burkholder A.B., Phillips B.T., Hall T.M.T. (2020). H/ACA snoRNA levels are regulated during stem cell differentiation. Nucleic Acids Res..

[119] Zhao M., Hou Y., Du Y.E., Yang L., Qin Y., Peng M., Liu S., Wan X., Qiao Y., Zeng H. (2020). Drosha-independent miR-6778-5p strengthens gastric cancer stem cell stemness via regulation of cytosolic one-carbon folate metabolism. Cancer Lett..

[120] Li Q., Yao Y., Eades G., Liu Z., Zhang Y., Zhou Q. (2014). Downregulation of miR-140 promotes cancer stem cell formation in basal-like early stage breast cancer. Oncogene.

[121] Butkytė S., Čiupas L., Jakubauskienė E., Vilys L., Mocevicius P., Kanopka A., Vilkaitis G. (2016). Splicing-dependent expression of microRNAs of mirtron origin in human digestive and excretory system cancer cells. Clin. Epigenetics.

[122] Mourksi N.E., Morin C., Fenouil T., Diaz J.J., Marcel V. (2020). snoRNAs Offer Novel Insight and Promising Perspectives for Lung Cancer Understanding and Management. Cells.

[123] Zhu W., Niu J., He M., Zhang L., Lv X., Liu F., Jiang L., Zhang J., Yu Z., Zhao L. (2019). SNORD89 promotes stemness phenotype of ovarian cancer cells by regulating Notch1-c-Myc pathway. J. Transl. Med..

[124] Yoshida K., Toden S., Weng W., Shigeyasu K., Miyoshi J., Turner J., Nagasaka T., Ma Y., Takayama T., Fujiwara T. (2017). SNORA21—An Oncogenic Small Nucleolar RNA, with a Prognostic Biomarker Potential in Human Colorectal Cancer. EBioMedicine.

[125] Abdelfattah A.M., Park C., Choi M.Y. (2014). Update on non-canonical microRNAs. Biomol. Concepts.

[126] Lakhotia S.C., Mallick B., Roy J., Pandey R. (2020). Chapter 2—Non-coding RNAs: Ever-expanding diversity of types and functions. Translational Epigenetics, Rna-Based Regulation in Human Health and Disease.

[127] Berezikov E., Chung W., Willis J., Cuppen E., Lai E.C. (2007). Mammalian Mirtron Genes. Mol. Cell.

[128] Ladewig E., Okamura K., Flynt A.S., Westholm J.O., Lai E.C. (2012). Discovery of hundreds of mirtrons in mouse and human small RNA data. Genome Res..

[129] Rorbach G., Unold O., Konopka B.M. (2018). Distinguishing mirtrons from canonical miRNAs with data exploration and machine learning methods. Sci. Rep..

[130] Kock K.H., Kong K.W., Hoon S., Seow Y. (2015). Functional VEGFA knockdown with artificial 3′-tailed mirtrons defined by 5′ splice site and branch point. Nucleic Acids Res..

[131] Wen J., Ladewig E., Shenker S., Mohammed J., Lai E.C. (2015). Analysis of Nearly One Thousand Mammalian Mirtrons Reveals Novel Features of Dicer Substrates. PLoS Comput. Biol..

[132] Sheng P., Fields C., Aadland K., Wei T., Kolaczkowski O., Gu T., Kolaczkowski B., Xie M. (2018). Dicer cleaves 5’-extended microRNA precursors originating from RNA polymerase II transcription start sites. Nucleic Acids Res..

[133] Castellano L., Stebbing J. (2013). Deep sequencing of small RNAs identifies canonical and non-canonical miRNA and endogenous siRNAs in mammalian somatic tissues. Nucleic Acids Res..

[134] Kim Y.K., Kim B., Kim V.N. (2016). Re-evaluation of the roles of DROSHA, Export in 5, and DICER in microRNA biogenesis. Proc. Natl. Acad. Sci. USA.

[135] Ruby J.G., Jan C.H., Bartel D.P. (2007). Intronic microRNA precursors that bypass Drosha processing. Nature.

[136] Scott M.S., Ono M. (2011). From snoRNA to miRNA: Dual function regulatory non-coding RNAs. Biochimie.

[137] Ender C., Krek A., Friedländer M.R., Beitzinger M., Weinmann L., Chen W., Pfeffer S., Rajewsky N., Meister G. (2008). A human snoRNA with microRNA-like functions. Mol. Cell.

[138] Li W., Saraiya A.A., Wang C.C. (2011). Gene regulation in Giardia lambia involves a putative microRNA derived from a small nucleolar RNA. PLoS Negl. Trop. Dis..

[139] Anderson P., Ivanov P. (2014). tRNA Fragments in Human Health and Disease. FEBS Lett..

[140] Shigematsu M., Honda S., Kirino Y. (2014). Transfer RNA as a Source of Small Functional RNA. J. Mol. Biol. Mol. Imaging.

[141] Maute R.L., Schneider C., Sumazin P., Holmes A., Califano A., Basso K., Dalla-Favera R. (2013). tRNA-derived microRNA modulates proliferation and the DNA damage response and is down-regulated in B cell lymphoma. Proc. Natl. Acad. Sci. USA.

[142] Krishna S., Yim D.G., Lakshmanan V., Tirumalai V., Koh J.L., Park J.E., Cheong J.K., Low J.L., Lim M.J., Sze S.K. (2019). Dynamic expression of tRNA-derived small RNAs define cellular states. EMBO Rep..

[143] Magee R.G., Telonis A.G., Loher P., Londin E., Rigoutsos I. (2018). Profiles of miRNA Isoforms and tRNA Fragments in Prostate Cancer. Sci. Rep..

[144] Liu J., Valencia-Sanchez M.A., Hannon G.J., Parker R. (2005). MicroRNA-dependent localization of targeted mRNAs to mammalian P-bodies. Nat. Cell Biol..

[145] Liu J., Rivas F.V., Wohlschlegel J., Yates J.R., Parker R., Hannon G.J. (2005). A role for the P-body component GW182 in microRNA function. Nat. Cell Biol..

[146] Gagnon K.T., Li L., Chu Y., Janowski B.A., Corey D.R. (2014). RNAi factors are present and active in human cell nuclei. Cell Rep..

[147] Ohrt T., Mütze J., Staroske W., Weinmann L., Höck J., Crell K., Meister G., Schwille P. (2008). Fluorescence correlation spectroscopy and fluorescence cross-correlation spectroscopy reveal the cytoplasmic origination of loaded nuclear RISC in vivo in human cells. Nucleic Acids Res..

[148] Catalanotto C., Cogoni C., Zardo G. (2016). MicroRNA in Control of Gene Expression: An Overview of Nuclear Functions. Int. J. Mol. Sci.

[149] Roberts T.C. (2014). The MicroRNA Biology of the Mammalian Nucleus. Mol. Ther. Nucleic Acids.

[150] Stavast C.J., Erkeland S.J. (2019). The Non-Canonical Aspects of MicroRNAs: Many Roads to Gene Regulation. Cells.

[151] Castanotto D., Lingeman R., Riggs A.D., Rossi J.J. (2009). CRM1 mediates nuclear-cytoplasmic shuttling of mature microRNAs. Proc. Natl. Acad. Sci. USA.

[152] Zisoulis D.G., Kai Z.S., Chang R.K., Pasquinelli A.E. (2012). Autoregulation of microRNA biogenesis by let-7 and Argonaute. Nature.

[153] Liang H., Zhang J., Zen K., Zhang C.Y., Chen X. (2013). Nuclear microRNAs and their unconventional role in regulating non-coding RNAs. Protein Cell.

[154] Toms D., Pan B., Bai Y., Li J. (2021). Small RNA sequencing reveals distinct nuclear microRNAs in pig granulosa cells during ovarian follicle growth. J. Ovarian Res..

[155] Cipolla G.A. (2014). A non-canonical landscape of the microRNA system. Front. Genet..

[156] Hwang H.W., Wentzel E.A., Mendell J.T. (2007). A hexanucleotide element directs microRNA nuclear import. Science.

[157] Kim D.H., Saetrom P., Snøve O., Rossi J.J. (2008). MicroRNA-directed transcriptional gene silencing in mammalian cells. Proc. Natl. Acad. Sci. USA.

[158] Tang R., Li L., Zhu D., Hou D., Cao T., Gu H., Zhang J., Chen J., Zhang C.Y., Zen K. (2012). Mouse miRNA-709 directly regulates miRNA-15a/16-1 biogenesis at the posttranscriptional level in the nucleus: Evidence for a microRNA hierarchy system. Cell Res..

[159] Földes-Papp Z., König K., Studier H., Bückle R., Breunig H.G., Uchugonova A., Kostner G.M. (2009). Trafficking of mature miRNA-122 into the nucleus of live liver cells. Curr. Pharm. Biotechnol..

[160] Jeffries C.D., Fried H.M., Perkins D.O. (2011). Nuclear and cytoplasmic localization of neural stem cell microRNAs. RNA.

[161] Seok H., Ham J., Jang E.S., Chi S.W. (2016). MicroRNA Target Recognition: Insights from Transcriptome-Wide Non-Canonical Interactions. Mol. Cells.

[162] Kozar I., Philippidou D., Margue C., Gay L.A., Renne R., Kreis S. (2021). Cross-Linking Ligation and Sequencing of Hybrids (qCLASH) Reveals an Unpredicted miRNA Targetome in Melanoma Cells. Cancers.

[163] Wang X. (2014). Composition of seed sequence is a major determinant of microRNA targeting patterns. Bioinformatics.

[164] Li S., Nguyen T.D., Nguyen T.L., Nguyen T.A. (2020). Mismatched and wobble base pairs govern primary microRNA processing by human Microprocessor. Nat. Commun..

[165] Loeb G.B., Khan A.A., Canner D., Hiatt J.B., Shendure J., Darnell R.B., Leslie C.S., Rudensky A.Y. (2012). Transcriptome-wide miR-155 binding map reveals widespread noncanonical microRNA targeting. Mol. Cell.

[166] Tay Y., Zhang J., Thomson A.M., Lim B., Rigoutsos I. (2008). MicroRNAs to Nanog, Oct4 and Sox2 coding regions modulate embryonic stem cell differentiation. Nature.

[167] Kim V.N. (2005). MicroRNA biogenesis: Coordinated cropping and dicing. Nat. Rev. Mol..

[168] Westholm J.O., Ladewig E., Okamura K., Robine N., Lai E.C. (2012). Common and distinct patterns of terminal modifications to mirtrons and canonical microRNAs. RNA.

[169] Wang Y., Soneson C., Malinowska A.L., Laski A., Ghosh S., Kanitz A., Gebert L.F.R., Robinson M.D., Hall J. (2021). MiR-CLIP reveals iso-miR selective regulation in the miR-124 targetome. Nucleic Acids Res..

[170] Hinton A., Hunter S.E., Afrikanova I., Jones G.A., Lopez A.D., Fogel G.B., Hayek A., King C.C. (2014). sRNA-seq analysis of human embryonic stem cells and definitive endoderm reveals differentially expressed microRNAs and novel IsomiRs with distinct targets. Stem Cells.

[171] Yuan Z., Ding S., Yan M., Zhu X., Liu L., Tan S., Jin Y., Sun Y., Li Y., Huang T. (2015). Variability of miRNA expression during the differentiation of human embryonic stem cells into retinal pigment epithelial cells. Gene.

[172] Lan C., Peng H., McGowan E.M., Hutvagner G., Li J. (2018). An isomiR expression panel based novel breast cancer classification approach using improved mutual information. BMC Med. Genom..

[173] Li S.C., Liao Y.L., Ho M.R., Tsai K.W., Lai C.H., Lin W.C. (2012). miRNA arm selection and isomiR distribution in gastric cancer. BMC Genom..

[174] Kim J., Orkin S.H. (2011). Embryonic stem cell-specific signatures in cancer: Insights into genomic regulatory networks and implications for medicine. Genome Med..

[175] Kim J., Woo A.J., Chu J., Snow J.W., Fujiwara Y., Kim C.G., Cantor A.B., Orkin S.H. (2010). A Myc network accounts for similarities between embryonic stem and cancer cell transcription programs. Cell.

[176] Somervaille T.C., Matheny C.J., Spencer G.J., Iwasaki M., Rinn J.L., Witten D.M., Chang H.Y., Shurtleff S.A., Downing J.R., Cleary M.L. (2009). Hierarchical maintenance of MLL myeloid leukemia stem cells employs a transcriptional program shared with embryonic rather than adult stem cells. Cell Stem Cell.

[177] Güler G., Acikgoz E., Yavasoglu N.Ü.K., Bakan B., Goormaghtigh E., Aktug H. (2018). Deciphering the biochemical similarities and differences among mouse embryonic stem cells, somatic and cancer cells using ATR-FTIR spectroscopy. Analyst.

[178] Ben-Porath I., Thomson M.W., Carey V.J., Ge R., Bell G.W., Regev A., Weinberg R.A. (2008). An embryonic stem cell-like gene expression signature in poorly differentiated aggressive human tumors. Nat. Genet..

[179] Siegers G.M., Dutta I., Kang E.Y., Huang J., Köbel M., Postovit L.M. (2020). Aberrantly Expressed Embryonic Protein NODAL Alters Breast Cancer Cell Susceptibility to γδ T Cell Cytotoxicity. Front. Immunol..

[180] Mulas C., Kalkan T., Smith A. (2017). NODAL Secures Pluripotency upon Embryonic Stem Cell Progression from the Ground State. Stem Cell Rep..

[181] Parisi S., Castaldo D., Piscitelli S., D’Ambrosio C., Divisato G., Passaro F., Avolio R., Castellucci A., Gianfico P., Masullo M. (2021). Identification of RNA-binding proteins that partner with Lin28a to regulate Dnmt3a expression. Sci. Rep..

[182] Yu J., Vodyanik M.A., Smuga-Otto K., Antosiewicz-Bourget J., Frane J.L., Tian S., Nie J., Jonsdottir G.A., Ruotti V., Stewart R. (2007). Induced pluripotent stem cell lines derived from human somatic cells. Science.

[183] Tsanov K.M., Pearson D.S., Wu Z., Han A., Triboulet R., Seligson M.T., Powers J.T., Osborne J.K., Kane S., Gygi S.P. (2017). LIN28 phosphorylation by MAPK/ERK couples signalling to the post-transcriptional control of pluripotency. Nat. Cell Biol..

[184] Tsialikas J., Romer-Seibert J. (2015). LIN28: Roles and regulation in development and beyond. Development.

[185] Wang H., Zhao Q., Deng K., Guo X., Xia J. (2016). Lin28: An emerging important oncogene connecting several aspects of cancer. Tumour Biol..

[186] Pan P., Chen T., Zhang Y., Qi Z., Qin J., Cui G., Guo X. (2018). LIN28A inhibits lysosome-associated membrane glycoprotein 1 protein expression in embryonic stem and bladder cancer cells. Mol. Med. Rep..

[187] O’Day E., Le M.T.N., Imai S., Tan S.M., Kirchner R., Arthanari H., Hofmann O., Wagner G., Lieberman J. (2015). An RNA-binding Protein, Lin28, Recognizes and Remodels G-quartets in the MicroRNAs (miRNAs) and mRNAs It Regulates. J. Biol. Chem..

[188] Chen Y., Xie C., Zheng X., Nie X., Wang Z., Liu H., Zhao Y. (2019). LIN28/let-7/PD-L1 Pathway as a Target for Cancer Immunotherapy. Cancer Immunol. Res..

[189] Yin J., Zhao J., Hu W., Yang G., Yu H., Wang R., Wang L., Zhang G., Fu W., Dai L. (2017). Disturbance of the let-7/LIN28 double-negative feedback loop is associated with radio- and chemo-resistance in non-small cell lung cancer. PLoS ONE.

[190] Degrauwe N., Schlumpf T.B., Janiszewska M., Martin P., Cauderay A., Provero P., Riggi N., Suvà M.L., Paro R., Stamenkovic I. (2016). The RNA Binding Protein IMP2 Preserves Glioblastoma Stem Cells by Preventing let-7 Target Gene Silencing. Cell Rep..

[191] Lovnicki J., Gan Y., Feng T., Li Y., Xie N., Ho C.H., Lee A.R., Chen X., Nappi L., Han B. (2020). LIN28B promotes the development of neuroendocrine prostate cancer. J. Clin. Investig..

[192] Parisi S., Piscitelli S., Passaro F., Russo T. (2020). HMGA Proteins in Stemness and Differentiation of Embryonic and Adult Stem Cells. Int. J. Mol. Sci..

[193] Cui H., Song R., Wu J., Wang W., Chen X., Yin J. (2018). MicroRNA-337 regulates the PI3K/AKT and Wnt/β-catenin signaling pathways to inhibit hepatocellular carcinoma progression by targeting high-mobility group AT-hook 2. Am. J. Cancer Res..

[194] Zhang H., Wu X., Sui Z., Ma Z., Gong L., Meng B., Tang P., Yu Z. (2021). High-mobility group AT-hook 2 promotes growth and metastasis and is regulated by miR-204-5p in oesophageal squamous cell carcinoma. Eur. J. Clin. Investig..

[195] Jiao D., Liu Y., Tian Z. (2019). microRNA-493 inhibits tongue squamous cell carcinoma oncogenicity via directly targeting HMGA2. OncoTargets Ther..

[196] Tong H., Zhuang X., Cai J., Ding Y., Si Y., Zhang H., Shen M. (2019). Long noncoding RNA ZFAS1 promotes progression of papillary thyroid carcinoma by sponging miR-590-3p and upregulating HMGA2 expression. OncoTargets Ther..

[197] Malek A., Bakhidze E., Noske A., Sers C., Aigner A., Schäfer R., Tchernitsa O. (2008). HMGA2 gene is a promising target for ovarian cancer silencing therapy. Int. J. Cancer.

[198] Tan L., Wei X., Zheng L., Zeng J., Liu H., Yang S., Tan H. (2016). Amplified HMGA2 promotes cell growth by regulating Akt pathway in AML. J. Cancer Res. Clin. Oncol..

[199] Mansoori B., Duijf P.H.G., Mohammadi A., Najafi S., Roshani E., Shanehbandi D., Hajiasgharzadeh K., Shirjang S., Ditzel H.J., Kazemi T. (2020). Overexpression of HMGA2 in breast cancer promotes cell proliferation, migration, invasion and stemness. Expert Opin. Ther. Targets.

[200] Esmailzadeh S., Mansoori B., Mohammadi A., Shanehbandi D., Baradaran B. (2017). siRNA-mediated silencing of HMGA2 induces apoptosis and cell cycle arrest in human colorectal carcinoma. J. Gastrointest. Cancer.

[201] Mansoori B., Mohammadi A., Ditzel H.J., Duijf P.H.G., Khaze V., Gjerstorff M.F., Baradaran B. (2021). HMGA2 as a Critical Regulator in Cancer Development. Genes.

[202] Mansoori B., Duijf P.H.G., Mohammadi A., Safarzadeh E., Ditzel H.J., Gjerstorff M.F., Cho W.C., Baradaran B. (2021). MiR-142-3p targets HMGA2 and suppresses breast cancer malignancy. Life Sci..

[203] Xu X., Zou H., Luo L., Wang X., Wang G. (2019). MicroRNA-9 exerts antitumor effects on hepatocellular carcinoma progression by targeting HMGA2. FEBS Open Bio.

[204] Chen X., Zeng K., Xu M., Liu X., Hu X., Xu T., He B., Pan Y., Sun H., Wang S. (2019). P53-induced miR-1249 inhibits tumor growth, metastasis, and angiogenesis by targeting VEGFA and HMGA2. Cell Death Dis..

[205] Mei L.L., Wang W.J., Qiu Y.T., Xie X.F., Bai J., Shi Z.Z. (2017). miR-125b-5p functions as a tumor suppressor gene partially by regulating HMGA2 in esophageal squamous cell carcinoma. PLoS ONE.

[206] Unachukwu U., Chada K., D’Armiento J. (2020). High Mobility Group AT-Hook 2 (HMGA2) Oncogenicity in Mesenchymal and Epithelial Neoplasia. Int. J. Mol. Sci..

[207] Yin J., Kim T.H., Park N., Shin D., Choi H.I., Cho S., Park J.B., Kim J.H. (2016). TRIM71 suppresses tumorigenesis via modulation of Lin28B-let-7-HMGA2 signaling. Oncotarget.

[208] Zhang K., Gao H., Wu X., Wang J., Zhou W., Sun G., Wang J., Wang Y., Mu B., Kim C. (2014). Frequent overexpression of HMGA2 in human atypical teratoid/rhabdoid tumor and its correlation with let-7a3/let-7b miRNA. Clin. Cancer Res..

[209] Lee Y.S., Dutta A. (2007). The tumor suppressor microRNA let-7 represses the HMGA2 oncogene. Genes Dev..

[210] Klemke M., Meyer A., Nezhad M.H., Belge G., Bartnitzke S., Bullerdiek J. (2010). Loss of let-7 binding sites resulting from truncations of the 3′ untranslated region of HMGA2 mRNA in uterine leiomyomas. Cancer Genet. Cytogenet..

[211] Chen T., You Y., Jiang H., Wang Z.Z. (2017). Epithelial-mesenchymal transition (EMT): A biological process in the development, stem cell differentiation, and tumorigenesis. J. Cell Physiol..

[212] Katsuno Y., Derynck R. (2021). Epithelial plasticity, epithelial-mesenchymal transition, and the TGF-β family. Dev. Cell.

[213] Vasaikar S.V., Deshmukh A.P., den Hollander P., Addanki S., Kuburich N.A., Kudaravalli S., Joseph R., Chang J.T., Soundararajan R., Mani S.A. (2021). EMTome: A resource for pan-cancer analysis of epithelial-mesenchymal transition genes and signatures. Br. J. Cancer.

[214] Thiery J.P., Acloque H., Huang R.Y., Nieto M.A. (2009). Epithelial-mesenchymal transitions in development and disease. Cell.

[215] Cesaro E., Pastore A., Polverino A., Manna L., Divisato G., Quintavalle C., Di Sanzo M., Faniello M.C., Grosso M., Costanzo P. (2021). ZNF224 is a mediator of TGF-β pro-oncogenic function in melanoma. Hum. Mol. Genet..

[216] Abba M.L., Patil N., Leupold J.H., Allgayer H. (2016). MicroRNA Regulation of Epithelial to Mesenchymal Transition. J. Clin. Med..

[217] Díaz-López A., Moreno-Bueno G., Cano A. (2014). Role of microRNA in epithelial to mesenchymal transition and metastasis and clinical perspectives. Cancer Manag. Res..

[218] Fontana A., Barbano R., Dama E., Pasculli B., Rendina M., Morritti M.G., Melocchi V., Castelvetere M., Valori V.M., Ravaioli S. (2021). Combined analysis of miR-200 family and its significance for breast cancer. Sci. Rep..

[219] Bortolin-Cavaille M.L., Dance M., Weber M., Cavaille J. (2009). C19MC microRNAs are processed from introns of large Pol-II, non-protein-coding transcripts. Nucleic Acids Res..

[220] Nguyen P.N., Huang C.J., Sugii S., Cheong S.K., Choo K.B. (2017). Selective activation of miRNAs of the primate-specific chromosome 19 miRNA cluster (C19MC) in cancer and stem cells and possible contribution to regulation of apoptosis. J. Biomed. Sci..

[221] Rippe V., Dittberner L., Lorenz V.N., Drieschner N., Nimzyk R., Sendt W., Junker K., Belge G., Bullerdiek J. (2010). The two stem cell microRNA gene clusters C19MC and miR-371-3 are activated by specific chromosomal rearrangements in a subgroup of thyroid adenomas. PLoS ONE.

[222] Tan E.J., Kahata K., Idås O., Thuault S., Heldin C.H., Moustakas A. (2015). The high mobility group A2 protein epigenetically silences the Cdh1 gene during epithelial-to-mesenchymal transition. Nucleic Acids Res..

[223] Ma J., Kong F., Yang D., Yang H., Wang C., Cong R., Ma X. (2021). lncRNA MIR210HG promotes the progression of endometrial cancer by sponging miR-337-3p/137 via the HMGA2-TGF-β/Wnt pathway. Mol. Therapy-Nucleic Acids.

[224] Ala U. (2020). Competing Endogenous RNAs, Non-Coding RNAs and Diseases: An Intertwined Story. Cells.

[225] Liu Y., Xue M., Du S., Feng W., Zhang K., Zhang L., Liu H., Jia G., Wu L., Hu X. (2019). Competitive endogenous RNA is an intrinsic component of EMT regulatory circuits and modulates EMT. Nat. Commun..

[226] Ala U., Karreth F.A., Bosia C., Pagnani A., Taulli R., Léopold V., Tay Y., Provero P., Zecchina R., Pandolfi P.P. (2013). Integrated transcriptional and competitive endogenous RNA networks are cross-regulated in permissive molecular environments. Proc. Natl. Acad. Sci. USA.

[227] Wang Y., Xu Z., Jiang J., Xu C., Kang J., Xiao L., Wu M., Xiong J., Guo X., Liu H. (2013). Endogenous miRNA sponge lincRNA-RoR regulates Oct4, Nanog, and Sox2 in human embryonic stem cell self-renewal. Dev. Cell.

[228] Xie D., Tong M., Xia B., Feng G., Wang L., Li A., Luo G., Wan H., Zhang Z., Zhang H. (2021). Long noncoding RNA lnc-NAP sponges mmu-miR-139-5p to modulate Nanog functions in mouse ESCs and embryos. RNA Biol..

[229] Qi X., Zhang D.H., Wu N., Xiao J.H., Wang X., Ma W. (2015). ceRNA in cancer: Possible functions and clinical implications. J. Med. Genet..

[230] Chen W., Yang J., Fang H., Li L., Sun J. (2020). Relevance Function of Linc-ROR in the Pathogenesis of Cancer. Front. Cell Dev. Biol..

[231] Yan Z.Y., Sun X.C. (2018). LincRNA-ROR functions as a ceRNA to regulate Oct4, Sox2, and Nanog expression by sponging miR-145 and its effect on biologic characteristics of colonic cancer stem cells. Zhonghua Bing Li Xue Za Zhi.

[232] Li C., Lu L., Feng B., Zhang K., Han S., Hou D., Chen L., Chu X., Wang R. (2017). The lincRNA-ROR/miR-145 axis promotes invasion and metastasis in hepatocellular carcinoma via induction of epithelial-mesenchymal transition by targeting ZEB2. Sci. Rep..

[233] Mi Y., Li Y., He Z., Chen D., Hong Q., You J. (2021). Upregulation of Linc-ROR Promotes the Proliferation, Migration, and Invasion of Gastric Cancer Cells Through miR-212-3p/FGF7 Axis. Cancer Manag. Res..

[234] Fu Z., Li G., Li Z., Wang Y., Zhao Y., Zheng S., Ye H., Luo Y., Zhao X., Wei L. (2017). Endogenous miRNA Sponge LincRNA-ROR promotes proliferation, invasion and stem cell-like phenotype of pancreatic cancer cells. Cell Death Discov..

[235] Gao Y., Luo X., Zhang J. (2021). LincRNA-ROR is activated by H3K27 acetylation and induces EMT in retinoblastoma by acting as a sponge of miR-32 to activate the Notch signaling pathway. Cancer Gene Ther..

[236] Jonkers I., Monkhorst K., Rentmeester E., Grootegoed J.A., Grosveld F., Gribnau J. (2008). Xist RNA is confined to the nuclear territory of the silenced X chromosome throughout the cell cycle. Mol. Cell Biol..

[237] Zeng Z.L., Lu J.H., Wang Y., Sheng H., Wang Y.N., Chen Z.H., Wu Q.N., Zheng J.B., Chen Y.X., Yang D.D. (2021). The lncRNA XIST/miR-125b-2-3p axis modulates cell proliferation and chemotherapeutic sensitivity via targeting Wee1 in colorectal cancer. Cancer Med..

[238] O’Brien S.J., Bishop C., Hallion J., Fiechter C., Scheurlen K., Paas M., Burton J., Galandiuk S. (2020). Long non-coding RNA (lncRNA) and epithelial-mesenchymal transition (EMT) in colorectal cancer: A systematic review. Cancer Biol. Ther..

[239] Wu X.B., Feng X., Chang Q.M., Zhang C.W., Wang Z.F., Liu J., Hu Z.Q., Liu J.Z., Wu W.D., Zhang Z.P. (2019). Cross-talk among AFAP1-AS1, ACVR1 and microRNA-384 regulates the stemness of pancreatic cancer cells and tumorigenicity in nude mice. J. Exp. Clin. Cancer Res..

[240] Cheng F.H.C., Lin H.Y., Hwang T.W., Chen Y.C., Huang R.L., Chang C.B., Yang W., Lin R.I., Lin C.W., Chen G.C.W. (2019). E2F6 functions as a competing endogenous RNA, and transcriptional repressor, to promote ovarian cancer stemness. Cancer Sci..

[241] Li M., Chai H.F., Peng F., Meng Y.T., Zhang L.Z., Zhang L., Zou H., Liang Q.L., Li M.M., Mao K.G. (2018). Estrogen receptor β upregulated by lncRNA-H19 to promote cancer stem-like properties in papillary thyroid carcinoma. Cell Death Dis..

[242] Peng F., Wang J.H., Fan W.J., Meng Y.T., Li M.M., Li T.T., Cui B., Wang H.F., Zhao Y., An F. (2018). Glycolysis gatekeeper PDK1 reprograms breast cancer stem cells under hypoxia. Oncogene.

[243] Zhou J., Qiu C., Fan Z., Liu T., Liu T. (2021). Circular RNAs in stem cell differentiation: A sponge-like role for miRNAs. Int. J. Med. Sci..

[244] Pascale E., Divisato G., Palladino R., Auriemma M., Ngalya E.F., Caiazzo M. (2020). Noncoding RNAs and Midbrain DA Neurons: Novel Molecular Mechanisms and Therapeutic Targets in Health and Disease. Biomolecules.

[245] Memczak S., Jens M., Elefsinioti A., Torti F., Krueger J., Rybak A., Maier L., Mackowiak S.D., Gregersen L.H., Munschauer M. (2013). Circular RNAs are a large class of animal RNAs with regulatory potency. Nature.

[246] Barilani M., Cherubini A., Peli V., Polveraccio F., Bollati V., Guffanti F., Del Gobbo A., Lavazza C., Giovanelli S., Elvassore N. (2020). A circular RNA map for human induced pluripotent stem cells of foetal origin. EBioMedicine.

[247] Lin Z., Tang X., Wan J., Zhang X., Liu C., Liu T. (2021). Functions and mechanisms of circular RNAs in regulating stem cell differentiation. RNA Biol..

[248] Izuogu O.G., Alhasan A.A., Mellough C., Collin J., Gallon R., Hyslop J., Mastrorosa F.K., Ehrmann I., Lako M., Elliott D.J. (2018). Analysis of human ES cell differentiation establishes that the dominant isoforms of the lncRNAs RMST and FIRRE are circular. BMC Genom..

[249] Yu C.Y., Li T.C., Wu Y.Y., Yeh C.H., Chiang W., Chuang C.Y., Kuo H.C. (2017). The circular RNA circBIRC6 participates in the molecular circuitry controlling human pluripotency. Nat. Commun..

[250] Kristensen L.S., Hansen T.B., Venø M.T., Kjems J. (2018). Circular RNAs in cancer: Opportunities and challenges in the field. Oncogene.

[251] Vo J.N., Cieslik M., Zhang Y., Shukla S., Xiao L., Zhang Y., Wu Y.M., Dhanasekaran S.M., Engelke C.G., Cao X. (2019). The Landscape of Circular RNA in Cancer. Cell.

[252] Rickert D., Bartl J., Picard D., Bernardi F., Qin N., Lovino M., Puget S., Meyer F.D., Mahoungou Koumba I., Beez T. (2021). Circular RNA profiling distinguishes medulloblastoma groups and shows aberrant RMST overexpression in WNT medulloblastoma. Acta Neuropathol..

[253] Yang H., Zhao M., Zhao L., Li P., Duan Y., Li G. (2020). CircRNA BIRC6 promotes non-small cell lung cancer cell progression by sponging microRNA-145. Cell. Oncol..

[254] Yang G., Wang X., Liu B., Lu Z., Xu Z., Xiu P., Liu Z., Li J. (2019). circ-BIRC6, a circular RNA, promotes hepatocellular carcinoma progression by targeting the miR-3918/Bcl2 axis. Cell Cycle.

[255] Wu Y., Zhang Y., Zheng X., Dai F., Lu Y., Dai L., Niu M., Guo H., Li W., Xue X. (2020). Circular RNA circCORO1C promotes laryngeal squamous cell carcinoma progression by modulating the let-7c-5p/PBX3 axis. Mol. Cancer.

[256] Yan N., Xu H., Zhang J., Xu L., Zhang Y., Zhang L., Xu Y., Zhang F. (2017). Circular RNA profile indicates circular RNA VRK1 is negatively related with breast cancer stem cells. Oncotarget.

[257] Rengganaten V., Huang C.J., Tsai P.H., Wang M.L., Yang Y.P., Lan Y.T., Fang W.L., Soo S., Ong H.T., Cheong S.K. (2020). Mapping a Circular RNA-microRNA-mRNA-Signaling Regulatory Axis That Modulates Stemness Properties of Cancer Stem Cell Populations in Colorectal Cancer Spheroid Cells. Int. J. Mol. Sci..

[258] Zhao J., Jiang Y., Zhang H., Zhou J., Chen L., Li H., Xu J., Zhang G., Jing Z. (2021). The SRSF1/circATP5B/miR-185-5p/HOXB5 feedback loop regulates the proliferation of glioma stem cells via the IL6-mediated JAK2/STAT3 signaling pathway. J. Exp. Clin. Cancer Res..

[259] Jiang Y., Zhou J., Zhao J., Zhang H., Li L., Li H., Chen L., Hu J., Zheng W., Jing Z. (2020). The U2AF2/circRNA ARF1/miR-342-3p/ISL2 feedback loop regulates angiogenesis in glioma stem cells. J. Exp. Clin. Cancer Res..

[260] Jiang X., Xing L., Chen Y., Qin R., Song S., Lu Y., Xie S., Wang L., Pu H., Gui X. (2020). CircMEG3 inhibits telomerase activity by reducing Cbf5 in human liver cancer stem cells. Mol. Ther. Nucleic Acids.

[261] Ambros V. (2004). The functions of animal microRNAs. Nature.

[262] Guo Z., Maki M., Ding R., Yang Y., Zhang B., Xiong L. (2014). Genome-wide survey of tissue-specific microRNA and transcription factor regulatory networks in 12 tissues. Sci. Rep..

[263] Landgraf P., Rusu M., Sheridan R., Sewer A., Iovino N., Aravin A., Pfeffer S., Rice A., Kamphorst A.O., Landthaler M. (2007). A mammalian microRNA expression atlas based on small RNA library sequencing. Cell.

[264] Ludwig N., Leidinger P., Becker K., Backes C., Fehlmann T., Pallasch C., Rheinheimer S., Meder B., Stähler C., Meese E. (2016). Distribution of miRNA expression across human tissues. Nucleic Acids Res..

[265] Lagos-Quintana M., Rauhut R., Yalcin A., Meyer J., Lendeckel W., Tuschl T. (2002). Identification of tissue-specific microRNAs from mouse. Curr. Biol..

[266] Peck B.C., Mah A.T., Pitman W.A., Ding S., Lund P.K., Sethupathy P. (2017). Functional Transcriptomics in Diverse Intestinal Epithelial Cell Types Reveals Robust MicroRNA Sensitivity in Intestinal Stem Cells to Microbial Status. J. Biol. Chem..

[267] Collino F., Deregibus M.C., Bruno S., Sterpone L., Aghemo G., Viltono L., Tetta C., Camussi G. (2010). Microvesicles derived from adult human bone marrow and tissue specific mesenchymal stem cells shuttle selected pattern of miRNAs. PLoS ONE.

[268] Rossi F., Noren H., Jove R., Beljanski V., Grinnemo K.H. (2020). Differences and similarities between cancer and somatic stem cells: Therapeutic implications. Stem Cell Res. Ther..

[269] Zhao X., Moore D.L. (2018). Neural stem cells: Developmental mechanisms and disease modeling. Cell Tissue Res..

[270] Diana A., Gaido G., Murtas D. (2019). MicroRNA Signature in Human Normal and Tumoral Neural Stem Cells. Int. J. Mol. Sci..

[271] Ciafrè S.A., Galardi S., Mangiola A., Ferracin M., Liu C.G., Sabatino G., Negrini M., Maira G., Croce C.M., Farace M.G. (2005). Extensive modulation of a set of microRNAs in primary glioblastoma. Biochem. Biophys. Res. Commun..

[272] Garg N., Vijayakumar T., Bakhshinyan D., Venugopal C., Singh S.K. (2015). MicroRNA Regulation of Brain Tumour Initiating Cells in Central Nervous System Tumours. Stem Cells Int..

[273] Chan J.A., Krichevsky A.M., Kosik K.S. (2005). MicroRNA-21 is an antiapoptotic factor in human glioblastoma cells. Cancer Res..

[274] Yamashita D., Kondo T., Ohue S., Takahashi H., Ishikawa M., Matoba R., Suehiro S., Kohno S., Harada H., Tanaka J. (2015). miR340 suppresses the stem-like cell function of glioma-initiating cells by targeting tissue plasminogen activator. Cancer Res..

[275] Floyd D.H., Zhang Y., Dey B.K., Kefas B., Breit H., Marks K., Dutta A., Herold-Mende C., Synowitz M., Glass R. (2014). Novel anti-apoptotic microRNAs 582-5p and 363 promote human glioblastoma stem cell survival via direct inhibition of caspase 3, caspase 9, and Bim. PLoS ONE.

[276] Papagiannakopoulos T., Friedmann-Morvinski D., Neveu P., Dugas J.C., Gill R.M., Huillard E., Liu C., Zong H., Rowitch D.H., Barres B.A. (2012). Pro-neural miR-128 is a glioma tumor suppressor that targets mitogenic kinases. Oncogene.

[277] Tomei S., Volontè A., Ravindran S., Mazzoleni S., Wang E., Galli R., Maccalli C. (2021). MicroRNA Expression Profile Distinguishes Glioblastoma Stem Cells from Differentiated Tumor Cells. J. Pers. Med..

[278] Mukherjee S., Paricio N., Sokol N.S. (2021). A stress-responsive miRNA regulates BMP signaling to maintain tissue homeostasis. Proc. Natl. Acad. Sci. USA.

[279] Sakaguchi M., Hisamori S., Oshima N., Sato F., Shimono Y., Sakai Y. (2016). miR-137 Regulates the Tumorigenicity of Colon Cancer Stem Cells through the Inhibition of DCLK1. Mol. Cancer Res..

[280] Shah M.S., Kim E., Davidson L.A., Knight J.M., Zoh R.S., Goldsby J.S., Callaway E.S., Zhou B., Ivanov I., Chapkin R.S. (2016). Comparative effects of diet and carcinogen on microRNA expression in the stem cell niche of the mouse colonic crypt. Biochim. Biophys. Acta.

[281] Wei R., Yang Q., Han B., Li Y., Yao K., Yang X., Chen Z., Yang S., Zhou J., Li M. (2017). microRNA-375 inhibits colorectal cancer cells proliferation by downregulating JAK2/STAT3 and MAP3K8/ERK signaling pathways. Oncotarget.

[282] Celià-Terrassa T. (2018). Mammary Stem Cells and Breast Cancer Stem Cells: Molecular Connections and Clinical Implications. Biomedicines.

[283] Pal B., Chen Y., Bert A., Hu Y., Sheridan J.M., Beck T., Shi W., Satterley K., Jamieson P., Goodall G.J. (2015). Integration of microRNA signatures of distinct mammary epithelial cell types with their gene expression and epigenetic portraits. Breast Cancer Res..

[284] Jinesh G.G., Flores E.R., Brohl A.S. (2018). Chromosome 19 miRNA cluster and CEBPB expression specifically mark and potentially drive triple negative breast cancers. PLoS ONE.

[285] Ward A., Shukla K., Balwierz A., Soons Z., König R., Sahin O., Wiemann S. (2014). MicroRNA-519a is a novel oncomir conferring tamoxifen resistance by targeting a network of tumour-suppressor genes in ER+ breast cancer. J. Pathol..

[286] Lv C., Li F., Li X., Tian Y., Zhang Y., Sheng X., Song Y., Meng Q., Yuan S., Luan L. (2020). Author Correction: MiR-31 promotes mammary stem cell expansion and breast tumorigenesis by suppressing Wnt signaling antagonists. Nat. Commun..

[287] Tordonato C., Marzi M.J., Giangreco G., Freddi S., Bonetti P., Tosoni D., Di Fiore P.P., Nicassio F. (2021). miR-146 connects stem cell identity with metabolism and pharmacological resistance in breast cancer. J. Cell Biol..

[288] Patel Y., Soni M., Awgulewitsch A., Kern M.J., Liu S., Shah N., Singh U.P., Chen H. (2019). Overexpression of miR-489 derails mammary hierarchy structure and inhibits HER2/neu-induced tumorigenesis. Oncogene.

[289] Ibarra I., Erlich Y., Muthuswamy S.K., Sachidanandam R., Hannon G.J. (2007). A role for microRNAs in maintenance of mouse mammary epithelial progenitor cells. Genes Dev..

[290] Plantamura I., Cataldo A., Cosentino G., Iorio M.V. (2020). miR-205 in Breast Cancer: State of the Art. Int. J. Mol. Sci..

[291] Song S.J., Poliseno L., Song M.S., Ala U., Webster K., Ng C., Beringer G., Brikbak N.J., Yuan X., Cantley L.C. (2013). MicroRNA-antagonism regulates breast cancer stemness and metastasis via TET-family-dependent chromatin remodeling. Cell.

[292] Bao C., Chen J., Chen D., Lu Y., Lou W., Ding B., Xu L., Fan W. (2020). MiR-93 suppresses tumorigenesis and enhances chemosensitivity of breast cancer via dual targeting E2F1 and CCND1. Cell Death Dis..

[293] Takashima Y., Terada M., Udono M., Miura S., Yamamoto J., Suzuki A. (2016). Suppression of lethal-7b and miR-125a/b Maturation by Lin28b Enables Maintenance of Stem Cell Properties in Hepatoblasts. Hepatology.

[294] De Menezes M.R., Acioli M.E.A., da Trindade A.C.L., da Silva S.P., de Lima R.E., da Silva Teixeira V.G., Vasconcelos L.R.S. (2021). Potential role of microRNAs as biomarkers in human glioblastoma: A mini systematic review from 2015 to 2020. Mol. Biol. Rep..

[295] Nana-Sinkam S.P., Croce C.M. (2011). MicroRNAs as therapeutic targets in cancer. Transl. Res..

[296] Zhu B., Ju S., Chu H., Shen X., Zhang Y., Luo X., Cong H. (2018). The potential function of microRNAs as biomarkers and therapeutic targets in multiple myeloma. Oncol Lett..

[297] Alvanegh A.G., Ganji S.M., Kamel A., Tavallaie M., Rafati A., Arpanaei A., Dorostkar R., Ghaleh H.E.G. (2021). Comparison of oncolytic virotherapy and nanotherapy as two new miRNA delivery approaches in lung cancer. Biomed. Pharmacother..

[298] Sempere L.F., Azmi A.S., Moore A. (2021). microRNA-based diagnostic and therapeutic applications in cancer medicine. Wiley Interdiscip. Rev. RNA.

